# GPA33 forms a distinct diagnostic target class to Claudin 18.2 in oesophageal adenocarcinoma enabling the development of a novel GPA33 antibody-based detection platform

**DOI:** 10.1186/s11658-025-00852-1

**Published:** 2026-03-10

**Authors:** Jack Brydon, Radovan Krejcir, Filip Zavadil-Kokas, Ashita Singh, Tomas Henek, Lenka Hernychova, Skye Coleman, Sofian Al Shboul, Vaclav Hrabal, Zuzana Kuncova, Marcos Yébenes Mayordomo, Łukasz Arcimowicz, Kathryn L. Ball, Monikaben Padariya, Umesh Kalathiya, Borivoj Vojtesek, Ted Hupp, J. R. O’Neill

**Affiliations:** 1 Institute of Genetics and Cancer (IGC), University of Edinburgh, Edinburgh, UK; 2 Research Centre for Applied Molecular Oncology, Masaryk Memorial Cancer Institute, Brno, Czech Republic; 3 Department of Pharmacology and Public Health, Faculty of Medicine, The Hashemite University, Zarqa, 13133 Jordan; 4 International Center for Cancer Vaccine Science (ICCVS), University of Gdansk, Gdansk, Poland; 5 Laboratory of Growth Regulators, Institute of Experimental Botany, The Czech Academy of Sciences, Olomouc, Czech Republic; 6 Cambridge Oesophagogastric Centre, Addenbrooke’s Hospital, Hills Rd, Cambridge, CB2 0QQ UK; 7 Department of Surgery, University of Cambridge, Cambridge, UK

**Keywords:** scFV, Phage biopanning, Epitope mapping, Immunohistochemistry, Oesophageal adenocarcinoma

## Abstract

**Background:**

Oesophageal adenocarcinoma (OAC) is a cancer of high unmet clinical need. Because of tumour heterogeneity, it is likely that OAC will be stratified into several subtypes. Claudin 18.2 antibodies form emerging novel therapeutics in patients with a subtype of OAC. A large-scale proteogenomics screen in OAC identified glycoprotein A33 (GPA33) protein as a dominating cancer-specific target. We set out to determine whether GPA33 is distinct from or overlaps with Claudin 18.2 as a theranostic target in OAC.

**Methods:**

A microarray from *n* = 106 patients, composed of cancer, normal squamous tissue, normal gastric tissue, and metastatic lymph nodes, was used to compare the expression of GPA33 and Claudin 18.2. A single-chain variable fragment (scFv)-phage display library was screened against recombinant GPA33 protein to isolate novel monoclonal antibodies. Next-generation complementarity-determining region 3 (CDR3) DNA sequencing (NGS) and enzyme-linked immunosorbent assay (ELISA) were both used to measure efficacy of antibody enrichment during biopanning.

**Results:**

GPA33 exhibits superior tumour-specific expression compared with Claudin 18.2, the latter of which is expressed in normal gastric tissue. GPA33 and Claudin 18.2 exhibit statistically significant mutually exclusive expression in cancer tissue cores; 36% of cancers are GPA33^+^/Claudin 18.2^−^, whilst 22% are GPA33^−^/Claudin 18.2^+^. GPA33 therefore forms a novel target for theranostics in a significant number of patients. A monoclonal antibody (RSE-05) targeting GPA33 was isolated from a scFV-phage display library. The antibody required a di-sulphide bridge to maintain its epitope on the antigen. Epitope mapping was performed using di-sulphide bridge mutagenesis, peptide-phage display, and XL-MS. The dominant epitope resides in the V-type IgG domain of GPA33 at residues 27–29 and structural amino acids S17 and K65. This di-sulphide bridge-constrained epitope defines a novel monoclonal antibody binding interface. The RSE-05 monoclonal antibody can be adapted and used as a capture-sensor tool to measure GPA33 protein in liquid phase using a two-site sandwich ELISA format.

**Conclusions:**

GPA33 exhibits elevated cancer-specific expression relative to Claudin 18.2, indicating that GPA33 can also form the basis for a cancer diagnostic. Claudin 18.2 and GPA33 generally exhibit mutually exclusive expression suggestive of two different OAC development pathways. Thus, GPA33 forms a novel target that captures the Claudin 18.2-negative patient class, and the monoclonal antibody we describe forms the basis for novel diagnostic and therapeutic tools for development in OAC.

**Supplementary Information:**

The online version contains supplementary material available at 10.1186/s11658-025-00852-1.

## Background

Oesophageal cancer is considered a cancer of high unmet clinical need owing to poor prognosis, late presentation, and the paucity of effective systemic therapies. There are two histological subtypes of oesophageal cancer: oesophageal squamous cell carcinoma (OSCC) and oesophageal adenocarcinoma (OAC) [1]. OSCC has a similar epidemiology to head and neck cancers, being linked to smoking, excessive alcohol consumption, and certain dietary components [2]. OAC develops through a multi-step process in response to refluxed bile and gastric acid leading to columnar metaplasia (Barrett’s oesophagus), which over time can progress to dysplasia and eventually adenocarcinoma [3]. Both OAC and Barrett’s oesophagus correlate closely with obesity, gastroesophageal reflux disease and male gender and worryingly continue to increase in incidence in Western countries [4].

Endoscopic treatment of dysplastic Barrett’s epithelium can prevent progression to OAC, and intervention for OAC at an early, asymptomatic, intramucosal stage carries a much more favourable prognosis than intervention on symptomatically detected cancer [5]. An effective screening test for the diagnosis of Barrett’s, therefore, remains a compelling objective, and non-endoscopic sponge-based technologies are undergoing evaluation [6]. The tumour suppressor p53 is integral in progression from Barrett’s to cancer, and *tp53* gene mutation undergoes a high rate of clonal selection in tissue during this process in tandem with *smad4* [7]. Biomarkers now exist for measuring progression through these pathological stages; therefore, surveillance and early detection is emerging as an important strategy to prevent OAC development [8]. Although there have been recent advances in the detection and surveillance of Barrett’s oesophagus using tailored biomarkers, the majority of patients with OAC still present with advanced disease and less than 20% will survive 5 years from diagnosis [9]. The cornerstone of current systemic therapy for patients with OAC is combination cytotoxic chemotherapy including platinum, anti-metabolite and taxane compounds [10]. Only a minority of patients exhibit a significant response to these drugs, and these are associated with off-target toxicity which limits their effectiveness [11].

Targeted therapies have been trialed in OAC with the aim of reducing toxicity. The most successful to date remains therapy directed against human epidermal growth factor receptor 2 (HER2)/erythroblastic oncogene B2 (ERBB2) with treatment using the monoclonal antibody trastuzumab in combination with chemotherapy resulting in an increase in mean overall survival relative to chemotherapy of 2.7 months [12]. In contrast, despite receptor amplification in 10–15% of patients, epidermal growth factor receptor (EGFR)-targeted therapies, using the monoclonal antibody panitumumab or gefitinib, either decreased overall survival or produced no clinical benefit for patients with advanced oesophageal cancer [13]. Programmed death-ligand 1 (PD-L1) antibodies have also emerged as a therapeutic option in oesophageal adenocarcinoma [14–16]. Finally, most recently monoclonal antibodies targeting the receptor protein Claudin 18.2 have shown promising effects alone or in combination with immunotherapies such as programmed cell death protein 1 (PD-1) or PD-L1 [17, 18]. Although some of these data on Claudin 18.2 demonstrate very modest impacts on survival, they provide a proof of concept that monoclonal antibodies can penetrate OAC tissue and lead to durable therapeutic responses.

Fully defining the complete extracellular receptor landscape of OAC may provide additional targets for monoclonal antibody-based therapy for evaluation. Towards this end, we previously carried out the largest multi-omics study in OAC aimed at identifying the most dominant pro-oncogenic membrane receptor or secreted oncoproteins for evaluation in monoclonal antibody diagnostics and/or therapeutic platforms [19, 20]. One of the compelling receptors identified at the protein level and found to be over-expressed in OAC relative to patient-matched adjacent normal tissues in ~ 30% of patients was GPA33. GPA33 was previously identified as over-expressed in Barrett’s epithelium and therefore provides both an option for screening applications and cancer therapeutics [21].

Although the mechanism of action of GPA33 in cancer is not well-defined, its promoter and expression appear active in intestinal cells [22]. GPA33 is also reported to be induced by peroxisome proliferator-activated receptor gamma (PPARγ) in a Krüppel-like factor 4 (KLF4)-dependent manner [23]. GPA33 is also correlated with Treg developmental stages [24] and is an epithelial marker of colon cancer [25]. Despite its unclear signalling action mechanism, monoclonal antibodies targeting this receptor have emerged as imaging tools [26], and possible bispecific therapeutics together with anti-cluster of differentiation 3 (CD3) antibodies in colorectal cancers [27]. With a potential role of GPA33 in both early detection of treatment of advanced OAC, we therefore sought to screen for monoclonal antibodies to GPA33 using a recently constructed synthetic phage antibody derived from canine patients along with using next-generation DNA sequencing methodologies to measure CDR3 sequence enrichment [28].

In this report, we compare the expression of GPA33 with the clinically emerging therapeutic target Claudin 18.2 to determine whether GPA33 stratification in cancer samples adds any potential value to therapeutics aimed at improving management of patients with OAC. We provide data indicating that GPA33 and Claudin 18.2 generally exhibit mutually exclusive expression in primary and metastatic lymph nodes. These data suggest that GPA33 acts as a biomarker to define a subclass of OAC not captured by Claudin 18.2. We also describe the generation of a monoclonal antibody that could form the basis for a future GPA33 theranostic, which has a novel, redox-regulated epitope that can be used to capture or sense GPA33 in a capture-detection liquid-phase diagnostic format. This forms the basis for future clinical evaluation of these biological tools in a lateral flow-based diagnostic device for rapid detection of pre-cancer/cancer lesions.

## Methods

### Antibodies and scFv-phage display

The GPA33 antibody described in this report was isolated from a scFV library using methods previously reported [28]. Human Fc-tagged GPA33 protein (Sino Biologics, cat. no. 11277-H08H) and His-tagged human GPA33 protein (Acro Biosystems, cat. no. GP3-H5224) were used for antibody selection. White 96-well high-protein binding immunoplates (Corning, cat. no. 3922) were used to capture antigens. Antigen (200 ng-1 µg) was coated into wells (100–200 µl) using sodium carbonate buffer (0.1 M, pH 9.0). After scFV phage library addition (whether parental library or phage pools) and washing to remove unbound scFV phage, the bound phage was eluted by incubation with 200 µl of 0.1 M triethylamine buffer (pH 10.0) for 20 min and neutralized using 70 µl of 0.1 M Tris–HCl buffer (pH 7.4). Phage was propagated in ER2735 cells (New England Biolabs, cat. no. E4104). The primary HRP-conjugated anti-M13 phage antibody (Santa Cruz, cat. no. sc-53004) was used to measure scFV phage binding (as in Figs. 2 and 3). A commercial anti-GPA33 rabbit polyclonal antibody (Sigma Atlas, cat. no. HPA018858) was used in ELISA or immunoblotting. The GPA33 antigen used to generate this polyclonal antibody was not disclosed but was stated to be derived from a protein epitope signature tag (prEST) antigen comprising 50–150 amino acids of the target protein that aims to minimize cross-reaction with other human proteins. A different GPA33 antibody was used for immunohistochemistry (Abcam, cat. no. ab108938), and the antigen used to generate this antibody is proprietary and was not disclosed. The RSE-05 monoclonal antibody formulation could not be used in immunohistochemistry of clinical samples (data not shown), presumably owing to epitope inaccessibility after antigen retrieval in the formalin-fixed paraffin tissue sections. Plasmids encoding synthetic anti-GPA33 scFV antibodies or IgG used mouse light chain and mouse heavy chain IgG2a constant domains, and such antibody encoding plasmids were obtained from TWIST Biosciences. Plasmid maps were generated using SnapGene software. Sequencing of scFv derived from colony isolation was performed by Source Bioscience (Cambridge, UK). A 12-mer peptide library was obtained from New England Biolabs for peptide-phage display (cat. no. E8111L). Antibody production in ExpiCHO cells using transfected plasmid DNA was carried out using the ExpiCHO mammalian expression system (Thermo Fisher) according to the manufacturer’s instructions. All chemicals were obtained from Sigma unless stated otherwise.

### Phage display epitope mapping

Buffers were filtered through a 0.22-μm filter before use. Protein G magnetic beads (Invitrogen, CA, USA) were washed three times with PBST and then diluted tenfold in the same buffer. Purified antibody (100 µg) was mixed with 100 µl of the bead suspension in a polypropylene 96-well plate and incubated with shaking for 1 h at room temperature. After coupling, the beads were washed twice with PBST containing 1 mg/ml BSA (PBST/BSA), transferred to a fresh plate, and washed once more in the same buffer. Beads were then resuspended in 100 µl PBST/BSA. The Ph.D.™-12 Phage Display Peptide Library was diluted 1:50 in PBST/BSA, and 100 µl of the diluted library (2 × 10^10^ pfu per sample) was added to the antibody-coated beads. Samples were incubated with shaking for 1 h at room temperature, followed by two washes in PBST and two washes in TBS (500 mM NaCl, 50 mM Tris, pH 8). The washed beads were resuspended in PBST, transferred to a clean plate, and washed in PBS containing 0.05% Tween-20. Bound phages were eluted with 50 µl of 0.1 M glycine (pH 3.0) and neutralized immediately using 8 µl of 1 M Tris (pH 8.0). The recovered phage DNA was amplified in three rounds of polymerase chain reaction (PCR) (for conditions and primers, see Ref. [29]) using Herculase II Fusion DNA Polymerase (Agilent, CA, USA). PCR products were size-selected and purified using SPRIselect magnetic beads (Beckman Coulter, IN, USA). The DNA library was sequenced using the Illumina Nextseq 550 system (Illumina, CA, USA). The reads derived from phage display samples underwent initial trimming, and unique 12 amino acid sequences were analysed. Subsequently, these sequences were aligned to the GPA33 protein sequence and presented graphically. In parallel, a comparable analysis was performed using the Hammock software [30], which employs a hidden Markov model-based clustering algorithm. The output was then visualized as a weblogo, highlighting a specific binding motif, if one was identified.

### ELISA assessment of GPA33 binding activity

For ELISA, 200 ng of target antigen was coated overnight per well of a 96-well plate in 50 µl of 0.1 M carbonate buffer (pH 9.2). Wells were washed twice with 150 µl of PBS-Tween-20 (0.1%, v/v) and then blocked with PBS-T-BSA (3% w/v) for 1 h. Blocking buffer was then removed, and 100 µl of antibody diluted in the blocking buffer was added to the wells. Antibodies being tested from ExpiCHO culture medium were diluted 1:4 in blocking buffer unless stated otherwise. Atlas pAb was diluted 1:1000 in blocking buffer. After a 1-h incubation, wells were washed four times with PBS-T as above. Anti-mouse HRP (for RSE-05, Agilent Technologies, cat. no. P026002) or anti-rabbit HRP (for Atlas pAb, Agilent Technologies, cat. no. P0217) was diluted 1:1000 in blocking buffer and 100 µl added per well to incubate for 1 h. Wells were washed four times as above, and 75 µl of enhanced chemiluminescence (ECL) solution added to each well and scanned for luminescence following 30 s of incubation using a Fluoroskan Ascent (PerkinElmer). For sandwich two-site ELISAs, antibodies were coated onto the plate as described above and blocked. GPA33 was diluted in PBS to the desired concentration, and 100 µl was added per well. Following 1 h incubation, wells were washed four times as above, and the ELISA then proceeded with addition of detection anti-GPA33 antibody as described above. For ELISAs using dithiothreitol (DTT) or sodium dodecyl sulfate (SDS) treatment of GPA33, treatment was performed as follows: Treatment agents (DTT and SDS) and GPA33 were each titrated to twice the desired final concentrations and then mixed in equal parts for single treatments. Where DTT and SDS were both being added, the three parts were titrated to three times the desired final concentration before mixing. These mixtures were then incubated for 10 min at 20 °C or 50 °C as indicated, before being diluted in carbonate coating buffer and used as above. For measuring relative *K*_d_, GPA33 was diluted to 4 mg/ml in 100 mM carbonate buffer (pH 9.0), and 50 µl was adsorbed per well of a white polystyrene 96-well plate overnight. Wells were washed twice with 150 ul of PBS-T (0.1% v/v). Wells were blocked with 120 µl of PBST-BSA (3% w/v) for 1 h of shaking. RSE-05 was diluted in this same blocking buffer by serial dilution. After removal of blocking buffer, 100 µl of diluted RSE-05 was added per well, and the plate shaken for 1 h. Wells were washed four times with 150 µl of PBST. Rabbit anti-mouse HRP was diluted 1:1000 in blocking buffer, and 100 µl was added to each well. Following a 1-h incubation, wells were washed four times with PBST, 75 µl of ECL was added per well, and luminescence was measured.

### Transfection of HEK293T cells with GPA33 and preparation of lysate

HEK293T cells were cultured in Dulbecco’s modified Eagle’s medium (DMEM; Sigma-Aldrich, cat. no. D5796) + 10% (v/v) foetal bovine serum (FBS), as adherent cultures. At 24 h prior to transfection, 3 × 10^5^ cells were seeded in 2 ml of media in six-well plates. Transfection mixtures were prepared by adding 2 μg of GPA33 expression plasmids and 6 μg of polyethylenimine (PEI; Polysciences Europe GmBH, cat. no. 23966-100) to 250 μl of DMEM, vortexing briefly, and then incubating at room temperature for 15 min. Mixtures were then added dropwise to each well, and cells were grown for a further 3 days. After 3 days, the medium was removed and the cell monolayer washed with ice-cold PBS. Cells were then detached and collected by scraping into 1 ml of ice-cold PBS and centrifuged at 1000 rpm for 10 min at 4 °C to pellet the cells. The PBS was removed, and 100 μl of IGEPAL lysis buffer was used to resuspend each pellet (1% IGEPAL (v/v), 150 mM NaCl, 50 mM Tris–HCl pH 8.0, 1 × protease inhibitor mix). This suspension was incubated on ice for 30 min, with brief vortexing at 10-min intervals. The resulting lysate was centrifuged at 10,000 rcf for 10 min, 4 °C, and the solubilized protein in the supernatant was collected and frozen. Alternatively, urea lysis buffer (8 M urea in PBS) was used for immunoblots, where similar data were acquired (data not shown).

### Immunoblotting of GPA33-containing lysates

Cys–Ser mutants of GPA33 were generated through site-directed mutagenesis. PCR reactions (50 μl) were set up as follows: 0.1 μM primers, 0.5 ng/μl GPA33 pcDNA (3.1) template plasmid, 1 × pfu master mix (Rovalab GmbH, cat. no. R550). Cycling conditions were: 95 °C for 2 min then 95 °C for 30 s, 65 °C for 30 s, and 72 °C for 2 min 30 s for 30 cycles, then 72 °C 5 m. Resultant PCR products were purified (Qiagen, cat. no. 28104, as directed) and quantified by NanoDrop. Template DNA was removed by digestion with DpnI (NEB, cat. no. R0176) at 37 °C for 15 m, prior to heat inactivation of DpnI by incubation at 80 °C for 20 m. Of this product, 5 μl was then transformed into chemically competent DH5a cells, which were plated onto LB-Agar-Amp plates and incubated overnight at 37 °C. Colonies from these plates were propagated in 5 ml LB-Amp liquid cultures overnight, and plasmids extracted (Qiagen, cat. no. 12125, as directed). Resultant plasmids were Sanger sequenced to confirm presence of Cys–Ser mutations. Primers were:

C43S F: 5′ CACCCTGCCCAGCACCTACCAC 3′; C43S R: 5′ GCTGGGCAGGGTGACACTCTTTC 3′; C96S F: 5′ CCTACGAGAGTTCTGTCTCG 3′; C96S R: 5′ GAACTCTCGTAGGTGCCGTTGTC 3′; C125S F: 5′ CAAACCAGAAAGCGGCATCGAG 3′; C125S R: 5′ CGCTTTCTGGTTTGGAGGGTGG 3′; C141S F: 5′ CAGCTGACCAGCCAATCAAAGG 3′; C141S R: 5′ GGCTGGTCAGCTGGATGTTGTTC 3’; C190S F: 5′ TTACTACATCAGTACCTCCAGC 3′; C190S R: 5′ GTACTGATGTAGTAACCCGATG 3′’; C201S F: 5′ CGCAGTTCAGCAACATCACGG 3′; and C201S R: 5′ TGCTGAACTGCGTCCCCTCCTC 3′. Plasmids were transfected into HEK293T, and lysates were used to access the effects of serine mutants on RSE-05 antibody binding. For SDS-polyacrylamide gel electrophoresis (PAGE) immunoblotting, 25 μg of protein for each sample was mixed with SDS sample buffer (± 50 mM DTT) before heating at 85 °C for 5 min. Samples were then separated by PAGE in a 10% acrylamide gel. Proteins were then transferred to the nitrocellulose membrane. For dot blots, protein samples were incubated at room temperature for 5 min ± 50 mM DTT before 12.5 μg was spotted onto nitrocellulose membrane and allowed to dry fully. Both blotting procedures then continued in the same fashion, with membranes being blocked with PBST-milk (5% w/v of milk and 0.1% Tween 20) for 1 h with gentle shaking. Membranes were then placed into primary antibody diluted in PBST-milk, either RSE-05 from ExpiCHO cell culture supernatant, diluted 1:4 unless stated otherwise, or Atlas pAb diluted 1:1000, and incubated for 1 h with gentle shaking. Membranes were then washed four times with PBST, for 5 min each wash. Secondary antibody (anti-mouse HRP or anti-rabbit HRP) was diluted 1:1000 in PBST-milk and added to membranes for 1 h. Membranes were washed with PBST as above. Blots were developed by addition of 1-step Ultra TMB blotting solution (Fisher Scientific, cat. no. 16375222), which was incubated until sufficient signal was observed. Within each experiment, membranes probed with the same primary antibody were incubated with TMB together and for the same length of time to ensure comparability. Membranes were then washed three times with water to stop development of signal and imaged immediately.

### Transfection of OAC cell panels with a GPA33 expression plasmid

FLO-1 (ECACC 11012001, grown with DMEM), OE33 (ECACC 96070808, grown with RPMI 1640), OE19 (ECACC 9607172, grown with RPMI 1640), OACP4 C (ECACC 11012005, grown with RPMI 1640), JH-EsoAd1 (RRID:CVCL_8098, grown with RPMI 1640) and HEK293T (ATCC, CRL-11268, grown with DMEM) cells were cultured in basal growth medium supplemented with 10% (v/v) foetal bovine serum and 1% (v/v) penicillin–streptomycin and maintained in monolayer adherent cultures at 37 °C with 5% CO_2_. DMEM is from Sigma-Aldrich (cat. no. D5796) and RPMI-1640 was from Sigma Aldrich (cat. no. R5886). At 24 h prior to plasmid transfection (as in Supplementary Fig. 4), 4 × 10^5^ cells were seeded in 2 ml of media in six-well plates using 0.05% trypsin/ethylenediamine tetraacetic acid (EDTA). Unless stated otherwise, transfection mixtures were prepared by adding 2 μg of the GPA233 expression plasmid or the control vector and 6μg of PEI into 250 μl of DMEM/RPMI, vortexing briefly, and then incubating at room temperature for 15 min. For co-transfections, plasmids were balanced to a complete plasmid mass of 2 ug (1:1 DNA ratio). Mixtures were then added dropwise to each well, and cells were grown for a further 48 h. After 48 h, the medium was removed and the cell monolayer washed with PBS. Cells were then detached and collected by scraping into 1.5 ml of PBS and centrifuged at 1200 rpm for 5 min at 4 °C to pellet the cells. The PBS was removed, and 100 ml of IGEPAL lysis buffer was used for each pellet. The lysis buffer includes: 1% IGEPAL (v/v), 150 mM NaCl, 50 mM Tris–HCl pH 8.0, 1 × bespoke protease inhibitor mixture, all without DTT. This suspension was incubated on ice for 20 min, the resulting lysate was centrifuged at 10,000 rpm for 10 min at 4 °C, and the solubilized protein in the supernatant was collected and stored at −20 °C. Proteins were quantified via a Bradford assay [31]. Total protein (30 mg) was mixed with SDS samples buffer without DTT (5% SDS (w/v), 20% glycerol (v/v), 0.02% bromophenol blue, 25 mM Tris–Hcl, pH 6.8, and then heated at 85 °C for 5 min. Proteins were resolved on a 12% acrylamide gel and subsequently transferred onto a nitrocellulose membrane. Membranes were blocked in 5% (w/v) milk in PBST for 10 min at room temperature with gentle shaking. The primary antibody to GPA33 (RSE-05) was prepared in PBST-milk and was applied to the membranes and incubated for 1 h with gentle shaking. Membranes were then washed three times with PBST (5 min per wash) and incubated for 1 h with the appropriate HRP-conjugated secondary antibody diluted in PBST-milk. After a final series of three 5-min PBST washes, membranes were incubated at room temperature with ECL solution for 5 min, and bound antibody was detected and imaged using the automated mode on the CYTIVA Image Quant 800 UV system.

Next-generation sequencing of CDR3 from antibody pools identify enriched antibody sequences was carried out as follows: Next-generation short-read DNA sequencing of rounds 1–4 from the GPA33 scFv phage biopanning screen and parental library was performed first by generating barcoded amplicons using PCR as recently reported [28]. The PCR primers are listed in Fig. 2A. The reverse primer containing the indexed barcode was 5′-GTG ACT GGA GTT CAG ACG TGT GCT CTT CCG ATCT XXX-FR4 sequences in Fig. 2A, where XXX is ACT (parental library), AAG (round 1 phage), TAT (round 2 phage), CAT (round 3 phage) and AAA (round 4 phage), where S in the primer is C or G. The forward primer 5′-ACACGACGCTCTTCCGATCTATGAAATACCTATTGCCTACGGCA-3′ codes for the PelB leader sequence at the N-terminus of the scFV was MKYLLPTA, followed by FR1 sequences. The PCR reaction included the forward primer and a pool of the four reverse primers (all at 1 µM concentrations in a final 25 µl volume), 1 µl of phage particles (~ 10^12^ pfu/ml), and Pfu DNA polymerase. After 35 cycles (63 °C primer hybridization and 72 °C elongation), the amplicons were processed on a 1.5% agarose gel in TAE buffer (Fig. 2D), and the DNA was excised and purified using a Qiagen amplicon isolation kit. The amplicon concentration ranged from 7 to 20 ng/µl in Tris–borate–EDTA (TBE) buffer. All amplicons were pooled into one reaction for processing by Source Bioscience (Cambridge UK). Source Bioscience performed quality control (2C), and the amplicon pool was blunt end ligated to the new generation of adaptor primers (NEB Next^®^ Ultra™ II for DNA Library Prep). The new adaptor sequences are as follows: adaptor read 1: 5′-AGATCGGAAGAGCACACGTCTGAACTCCAGTCA-3′; adaptor read 2: 5′-AGATCGGAAGAGCGTCGTGTAGGGAAAGAGTGT-3′. Indexed DNA sequencing reads passing quality control were extracted, and the sequences containing TGAGGAGACGGTGACCAGGGT after the triplet barcode tagging the heavy chain were extracted. DNA sequencing files were provided containing ~  > 1 M sequencing reads for the pooled amplicon library, and the top 10,000 sorted sequences are given in Supplementary Table 1.

### Flow cytometry

Cell cultures of MCF-7 (ATCC, number HTB-22) and MCF-7-GPA33 clones were trypsinized and washed with PBS. The cells were then blocked against unspecific binding with PBS containing 0.5% BSA for 15 min in 4 °C. Next, the cells were incubated with RSE-05 IgG for 1 h in 4 °C, followed by washing the samples with PBS twice. The samples were incubated with secondary antibody goat anti-mouse IgG coupled with Alexa Fluor™ 488 (Invitrogen, USA, cat. no. A32723) for 30 min at 4 °C. The cells were then washed twice with PBS and analysed using the BD FACSVerse™ (BD Biosciences, USA) instrument and BD FACSuite™ software (version 1.0.5, BD Biosciences, USA). MCF7 cell line expressing GPA33 protein was prepared by transfection with an expression vector and selection of stably transfected cells with the vector incorporated into their genome. A plasmid with GPA33 complementary DNA (cDNA) was purchased from Sino Biological, Inc. (Beijing, China), and the cDNA was cloned into an expression vector with a puromycin resistance gene using Gateway technology (Thermo Fisher Scientific, Waltham, USA). MCF7 cells were transfected using Lipofectamine™ 3000 transfection reagent (Thermo Fisher Scientific, cat. no. L3000008). After 24 h, the culture medium was exchanged for a new one with the addition of puromycin dihydrochloride (5 μg/ml) (GoldBio, St Louis, USA, cat. no. P-600-100), and the cells were incubated for 7 days. The pool of the selected cells was frozen for later use, and the single cells were sorted on a 96-well plate to produce clones using BD FACS Aria II cell sorter (BD Biosciences, Franklin Lakes, USA).

### Antibody production in the ExpiCHO system

RSE-05 was produced in the ExpiCHO system (Thermo Fisher Scientific, cat. no. A29127) according to the manufacturer’s instructions using the max titer protocol. Briefly, ExpiCHO cells were transfected using ExpiFectamine CHO transfection reagent and kept at 32 °C with 5% CO_2_ whilst shaking on an orbital shaker for 10–14 days until the cell viability decreased below 75%. After that, the supernatant was harvested by spinning down the cells, and the antibody was purified using a 1 ml HiTrap protein A FF column (Cytiva, MA, USA, cat. no. 17507901) and ÄKTA pure system (Cytiva). The supernatant was adjusted by adding Tris to 0.1 M, and pH was adjusted to 8. Then, the supernatant was passed through a 0.45-µm filter. The column was equilibrated with 0.1 M Tris pH 8, and the supernatant was loaded to the column. After that, the column was washed with 0.1 M Tris pH 8 and subsequently with 0.01 M Tris pH 8. Then, IgG was eluted with 0.1 M glycine pH 3, and the pH of the eluate was immediately neutralized with 1 M Tris pH 8. The elution buffer was exchanged to PBS pH 7.4 using a ZEBA spin column (Thermo Fisher Scientific, cat. no. 89893).

### Polyclonal antibody production for two-site ELISA

Two non-overlapping peptides derived from solvent accessible regions of GPA33 (Supplementary Fig. 6) were synthesized by PEPCEUTICALS BIOSYNTH Group (now BIOSYNTH B.V., The Netherlands). The anti GPA33 polyclonal antibodies to these regions of GPA33 were developed and purified by Moravian-Biotechnology spol. s r.o. (Brno, Czech Republic), who performed immunizations, serum collection and affinity purification (as in Fig. 9A, B). Polyclonal sera were produced in rabbits immunized with the synthetic peptides containing a N-terminal cysteine, conjugated to keyhole limpet hemocyanin at the N-terminal cysteine. Polyclonal rabbit serum was affinity purified using the same peptide conjugated to beads using SulfoLink immobilization (Thermo Scientific Pierce, MA, USA, cat. no. PI44999). As is standard practice in commercial polyclonal antibody generation, the peptide sequences used as antigens for antibody generation are not disclosed but the peptide sequences are available under a material transfer agreement (MTA) with the University of Edinburgh. The antigen sequences used for the other GPA33 antibodies (Sigma Atlas, cat. no. HPA018858; Abcam, cat. no. ab108938) have not been disclosed, and we did not epitope map these commercial antibodies using peptide phage display, as was done with RSE-05 (Fig. 7).

### Immunofluorescence microscopy of live cells

MCF7-GPA33 and MCF7 control cells were seeded on μ-Slide 8 Wells (Ibidi, Gräfelfing, Germany, cat. no. 80826,) at a density of 20 × 10^3^ cells per well and left to adhere for 24 h. After that, cells were washed in PBS followed by 15 min of incubation with a blocking solution (0.5% BSA in PBS). Cells were incubated with RSE-05 diluted to 2 μg/ml in the blocking solution for 1 h in the dark and washed twice with PBS. Then, cells were incubated with anti-mouse fluorescein isothiocyanate (FITC)-conjugated secondary antibody (Vector Laboratories Inc., Burlingame, CA, USA, cat. no. FI-2000-1.5) in the blocking solution for 30 min in the dark and washed twice in PBS. After that, cells were kept in the blocking solution and observed using a fluorescence microscope (Eclipse Ti-E, Nikon, Tokyo, Japan) under standard culture conditions (37 °C, 95% humidity, 5% CO_2_).

### Sample preparation for mass spectrometry

GPA33:RSE-05 cross-linked proteins were purified and concentrated via SDS-PAGE. Electrophoresis was performed at 80 V for 8 min, followed by staining with Coomassie Brilliant Blue G-250 (Supplementary Fig. 5B). Gel bands were excised, washed with deionized water, and diced into small fragments. Destaining to remove the Coomassie Blue dye was carried out using a 200 mM ammonium bicarbonate solution (NH_4_HCO_3_, pH 7.8) in 40% aqueous acetonitrile (ACN, v/v) at 30 °C for 20 min. The gel slices were then equilibrated in 50 mM NH_4_HCO_3_ (pH 7.8) containing 5% aqueous ACN (v/v) at 30 °C for 30 min. The supernatant was removed, and the gels were dehydrated with ACN. The supernatant was removed, and the samples were reduced by addition of 10 mM DTT at 60 °C for 60 min, followed by alkylation with 55 mM iodoacetamide in the dark for 45 min at room temperature. The supernatant was removed, and the gel slices were washed three times with an equilibration buffer and dehydrated with acetonitrile. Trypsin digestion was carried out at 37 °C overnight using Promega sequencing-grade trypsin (2 µg of trypsin in 100 µl of 50 mM hydrogen ammonium bicarbonate in 5% acetonitrile, pH 7.8). Digested peptides were extracted using ACN, vacuum dried, and desalted using C18 micro spin columns (Harvard Apparatus) according to the manufacturer’s guidelines. Before mass spectrometry analysis, the evaporated peptide samples were dissolved in 2% ACN (v/v) with 0.05% aqueous trifluoroacetic acid (v/v).

### Mass spectrometry of trypsinized GPA33:RSE-05 cross-linked protein mixtures

Liquid chromatography–tandem mass spectrometry (LC–MS/MS) analysis was performed with a timsTOF mass spectrometer (Bruker Daltonics) and with a captive spray nano electro-spray ionization (nanoESI) source (Bruker Daltonics) coupled to a NanoElute 2 liquid chromatograph unit. The peptides were loaded into an Acclaim PepMapTM 100 nano trap column (nanoViperTM C18, 0.3 × 5 mm, 5 µm particle size, 100 Å pore size; Thermo Fisher Scientific) using a mobile phase A (0.1% aqueous formic acid (v/v)) for 2 min to allow for desalting. The peptides were eluted (flow rate of 300 nl/min) onto an Aurora Ultimate C18 CSI (75 µm × 25 cm, 1.7 µm particle size, 120 Å pore size, IonOptics; an analytical column with integrated nanospray emitter, kept at 50 °C) using a linear gradient over 64 min from 2% to 25% B and 6-min gradient from 25% to 48% B, followed by a 5-min wash step with 95% B. Mobile phase B was acetonitrile 100% with 0.1% formic acid (v/v). The TOF mass analyser was operated in positive ion mode, a high sensitivity mode using a spray voltage of 1400 V, and the capillary was heated to 200 °C. The acquisition method was set to parallel accumulation, serial fragmentation (PASEF) using a ramp time of 100 ms with close to 100% duty cycle for a total of 1.18 s per PASEF cycle. The master scan was acquired within the mass range of 100–1700 *m*/*z* and a mobility range of 0.7–1.4 1/*K*_0_. Precursors for MS/MS were chosen at an intensity threshold of 1000. The dynamic exclusion time was set to 0.4 min. Precursor ions were then isolated with a 2-Th window for *m*/*z* < 700 and 3-Th for *m*/*z* > 700. The precursor ions were fragmented using collision-induced dissociation (CID) with collision energy as a linear ramp from 20 eV at 1/*K*_0_ = 0.6 V s cm^−2^ to 59 eV at 1/*K*_0_ = 1.6 V s cm^−2^. Spectra were analysed for DSS cross-linked adducts as previously reported [32]. Mass spectra were uploaded to PRIDE (dataset identifier PXD060098; username: reviewer_pxd060098@ebi.ac.uk; password: wTsh64h4DTRe).

### Immunohistochemistry (IHC) staining and evaluation

The tissue microarray (TMA) was constructed as previously reported [19, 20]. TMA blocks were sectioned at a thickness of 5 μm and placed on positively charged slides (PFM Medical UK Ltd, cat. no. S001-W-45) to maximize core adherence. Immunohistochemistry (IHC) was performed using the BOND III autostainer with antibodies to GPA33 (Abcam, cat. no. ab108938, titrated from 1:50 up to 1:500 to optimize signal to background) and Claudin 18.2 (Abcam, cat. no. ab241330, titrated from 1:50 up to 1:500 to optimize signal to background). The sections were incubated with the antibodies for 20 min at room temperature, followed by detection using the Leica Bond Polymer Refine Detection kit (Leica Biosystems, cat. no., DS9800). IHC staining of TMA cores was assessed as previously described [19, 20]. The TMA slides were scanned at 40× magnification using a NanoZoomer XR digital pathology slide scanner (Hamamatsu), and images were viewed using NDP.view 2 viewing software.

## Results

### Stratification of GPA33 expression in relation to Claudin 18.2 in an OAC tumour microarray

Our original aim in developing multiomics (including proteomics) analysis was to determine whether there were transmembrane receptors expressed in oesophageal adenocarcinoma (OAC) that could form the basis for monoclonal antibody-based diagnostics and therapeutics. Cancer tissue-specific receptor targets derived from the mass spectrometric data [19, 20] included several plasma transmembrane receptor targets. An important component of these proteomic studies was the inclusion of two normal adjacent tissue controls: normal oesophageal squamous mucosa and normal gastric epithelium [19, 20].

**Fig. 1:**
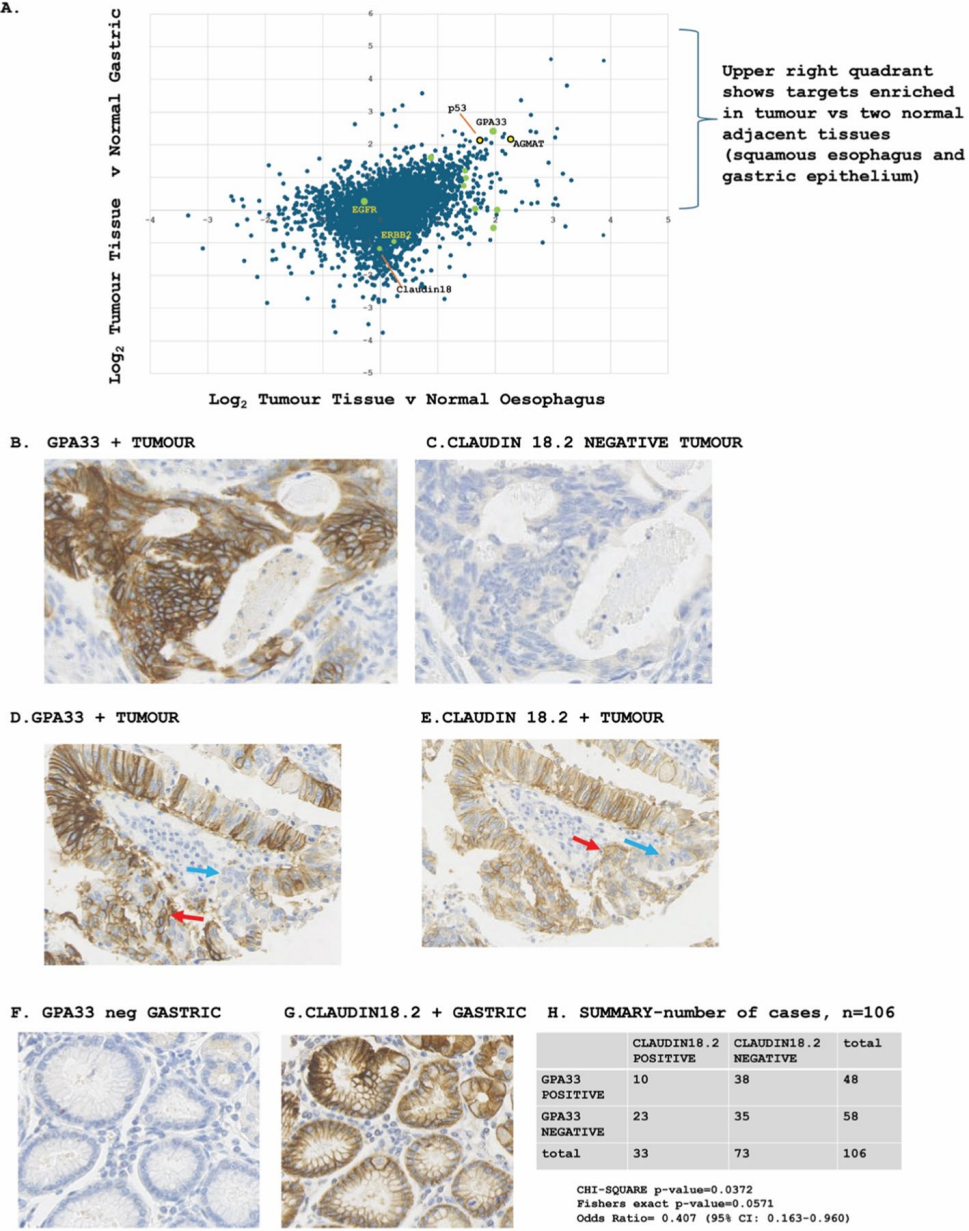
Expression of key transmembrane receptors in oesophageal adenocarcinoma. Identification of tumour specific transmembrane receptors in oesophageal adenocarcinoma. A previous study comprising the largest multi-OMIC characterization of oesophageal adenocarcinoma [20] applied mass spectrometry to matched tumour, normal adjacent oesophageal squamous mucosa and normal adjacent gastric mucosa to identify targets that were over-expressed in tumour relative to two related normal tissues. These data are plotted as the ratio of protein expression in tumour versus normal gastric and as a function of tumour versus normal oesophagus. We highlight GPA33 and Claudin 18, as well as other therapeutic antibody targets in OAC, including EGFR and ERBB2. The upper right quadrant contains the tumour elevated targets, where we highlight the transmembrane receptors that might form future diagnostic or therapeutic targets. We label this as Claudin 18 as the tryptic peptides did not cover the 18.2 isoform. **B**–**G** Representative expression of GPA33 and Claudin 18.2 in a *n* = 106 patient TMA. Serial slices of the TMA were taken to compare expression of GPA33 and Claudin 18.2 in the same tumour region. **B**, **C** A representative cancer that is GPA33 positive and Claudin 18.2 negative (*n* = 38 out of 106); **D**–**E** A representative cancer that is positive for GPA33 and Claudin 18.2 (*n* = 10 out of 106). Red and blue arrows in **D** and **E** highlight, within the same tumour field, membrane positive and membrane negative receptor expression, respectively. **F**, **G** A representative expression of GPA33 and Claudin 18.2 in normal gastric epithelium. The brown colour in the images reflects outer membrane expression. Positive GPA33 or Claudin 18.2 membrane expression in the cores used a similar inclusion criterion as described for PD-L1 using IHC, viz. partial or complete linear membrane staining (as described in pharmDx Interpretation Manual—Esophageal Cancer (Dako/Agilent). **H** A summary of the number of tumours (out of *n* = 106) with defined GPA33 and Claudin 18.2 expression status. The statistical data are as follows: chi-squared test (without correction): *χ*^2^ = 4.340, df = 1, *p* = 0.0372; Fisher’s exact test: *p* = 0.0571; odds ratio = 0.407 (95% confidence interval (CI) 0.163–0.960). The conclusion is that there is a negative association between GPA33 and Claudin 18.2 in cancer cores and their generally mutual exclusive expression is statistically significant

Several of these membrane targets might prove useful as potential diagnostics and/or therapeutics. However, GPA33 (Fig. 1A, green arrow, upper right quadrant) is compelling because it is not only a target for colorectal cancer therapeutics [27] but also a key biomarker commonly observed in oesophageal and gastric intestinal metaplasia [33]. GPA33 antibodies are generally being used for clinical approaches in the form of a CD3-bivalent antibody to stimulate T-cell dependent elimination of GPA33-positive colorectal cancers [34]. Radioimmunotherapy or theranostics is also being envisioned in preclinical models of GPA33-positive cancers [35], whereby the antibody is conjugated to radionuclides. Another target, agmatine ureohydrolase (AGMAT), is also shown in Fig. 1A, since this forms a class of protein like GPA33 in that it exhibits discordance in its protein-to-messenger RNA (mRNA) ratio [20]. P53 protein is also shown as an internal control (Fig. 1A), since the protein is highly mutated in OAC and therefore expressed at high levels in tumour tissue versus normal controls (Fig. 1A). A recently identified upper GI target, Claudin 18.2, is the focus of emerging monoclonal antibody (drug conjugated) medicines in gastric and upper GI junctional cancers [36, 37]. Claudin 18 tryptic peptides were also detected in our proteomics data, but this target did not exhibit enhanced tumour expression (Fig. 1A, red arrow). Since Claudin 18 does not exhibit tumour specific expression in our mass spectrometric data, one would normally exclude such targets for diagnostic or therapeutic developments. In fact, expression of Claudin 18.2 in normal gastric epithelium might be linked to gastric toxicity with anti-Claudin 18.2 antibody therapeutics [36]. Nevertheless, clinical research has shown that antibodies that bind to Claudin 18.2 can interact with this receptor on tumour cells [38]. As such, we first compared expression of Claudin 18.2 to GPA33 in patient-derived cancer tissue using immunohistochemistry because it was important to determine whether GPA33 and Claudin 18.2 are co-expressed, independently expressed or negatively correlated. For diagnostic and/or therapeutic purposes, if GPA33 was expressed in the same cancer cells as Claudin 18.2, it would be difficult to rationalize developing a new GPA33 therapeutic. Similarly, if GPA33 exhibited less cancer specificity than Claudin 18.2, then the value of GPA33 as a diagnostic would also be limited. A tumour microarray (TMA), which contained tissue cores including adenocarcinoma, normal adjacent squamous oesophagus, involved and non-involved nodes, and normal adjacent gastric tissue [20], was used to examine the extent of GPA33 and Claudin 18.2 expression in oesophageal cancers and normal adjacent tissue (gastric and oesophageal epithelium).

Using this TMA with *n* = 106 patients, we scored primary tumour cores for GPA33 and Claudin 18.2. A positive expression score for GPA33 or Claudin 18.2 required at least one ‘field’ of cancer cells showing membrane localization. Representative tumours with GPA33-positive ‘fields’ and Claudin 18.2-negative regions are shown in Fig. 1B, C. GPA33-positive expression at the membrane and GPA33-negative cells within the same core are highlighted (Fig. 1D, red and blue arrows, respectively). These fields of cells represent heterogeneous expression of the GPA33 receptor within the same cancer. GPA33 was usually heterogeneous within a primary tumour core, with another example of heterogeneity (i.e. GPA33-positive cells and GPA33-negative cells within the same patient core) shown in Supplementary Fig. 2A.

Representative tumours which have both GPA33-positive and Claudin 18.2-positive fields are shown in Fig. 1D, E, with Claudin 18.2 also showing its typical membrane localization. Claudin 18.2-positive expression in the membrane and Claudin 18.2 membrane-negative cells within the same tumour core are highlighted (Fig. 1E, red and blue arrows, respectively). Such cell fields represent heterogeneous expression of the Claudin 18.2 receptor within the same tumour. Dual expression of GPA33 and Claudin 18.2 occurred in 10 out of 106 patients (Fig. 1H). Representative dual expression of GPA33 and Claudin 18.2 is shown to suggest that, when the two are co-expressed, they appear to be in the same cells or cell fields (Fig. 1D, E). After quantitation of formalin-fixed paraffin-embedded (FFPE) cancer cores from *n* = 106 patients, the data demonstrate that GPA33^+^/Claudin 18.2^−^ expression comprised ~ 36% of tumours, relative to Claudin 18.2^+^/GPA33^−^, which comprised ~ 22% (Fig. 1H). The number of patient tumours that were negative for both receptors was ~ 33% (Fig. 1H). These data suggest that GPA33 is expressed in more patients with OAC than Claudin 18.2.

In multi-tissue analysis of the TMA (which includes normal adjacent squamous oesophagus, normal gastric epithelium and involved or non-involved lymph nodes), we find that Claudin 18.2 is expressed at relatively high levels on the membrane in normal gastric epithelia (representative image in Fig. 1G). Claudin 18.2 expression in normal gastric tissue has been noted [36]. the Human Protein Atlas also has data demonstrating that Claudin 18 is expressed in normal gastric tissue using three different antibodies, but whether these antibodies bind to the Claudin 18.2 isoform are not known. By contrast, GPA33 was not expressed at the membrane in normal gastric epithelia (representative image in Fig. 1F). The high Claudin 18.2 expression in normal gastric tissue is also consistent with the mass spectrometric data where Claudin 18 tryptic peptides were shown to be not tumour specific (Fig. 1A). These data suggest that GPA33 would be a superior cancer-specific biomarker compared with Claudin 18.2.

It is of interest to note that, unexpectedly, a large proportion of GPA33-positive or Claudin 18.2-positive tumours exhibited statistically significant, mutually exclusive expression in each patient’s cancer core (Fig. 1H). This is suggestive of there being two separate cancer developmental pathways or endpoint pathways that use these two receptors to drive or maintain the tumorigenic state (Supplementary Fig. 1). These data also suggest that two different therapeutics would be of value to target GPA33^+^ or Claudin 18.2^+^ tumours. As such, we also evaluated the expression of GPA33 and Claudin 18.2 in patient-matched, involved lymph node metastases (Supplementary Fig. 2). In concordance with primary tumours, we identified a largely mutually exclusive expression of GPA33 and Claudin 18.2 in the involved lymph nodes. A macro view of one representative immunostained TMA containing the lymph nodes, normal adjacent squamous oesophagus, and glandular gastric tissues, and adenocarcinoma is shown in Supplementary Fig. 3A, B. Representative images of a patient with GPA33-positive tumour and GPA33-positive involved lymph node, but no expression of Claudin 18.2 in the primary tumour or lymph node, are shown in Supplementary Fig. 2A ,B. Representative images of a patient with Claudin 18.2-positive tumour and Claudin 18.2-positive involved lymph node, but no expression of GPA33 in the primary tumour or lymph node, are shown in Supplementary Fig. 2C, D. Quantitation in Supplementary Fig. 2E reveals, again, a general trend towards mutually exclusive expression of both receptors in the involved lymph nodes. From *n* = 39 patients, 12 involved lymph nodes were GPA33-positive/Claudin 18.2-negative, 8 nodes were Claudin 18.2-positive/GPA33-negative and 17 nodes were dual-negative (Supplementary Fig. 2E). This is suggestive of two distinct OAC tumorigenic states (Supplementary Fig. 1). However, the mutually exclusive expression in the involved lymph node is not statistically significant (Supplementary Fig. 2E). Overall, these data demonstrate that GPA33 is over-expressed in a higher prevalence of patients than Claudin 18.2 with greater tumour specificity relative to adjacent normal tissues. Their expression also exhibits statistically significant mutual exclusivity in the primary tumour (Fig. 1H) with a similar trend in the involved lymph nodes (Supplementary Fig. 2). There may , therefore, be significant value in developing a GPA33 diagnostic and/or therapeutic because it captures a distinct patient group than the Claudin 18.2-positive patients’ cancers.

### Isolation of an anti-GPA33 scFv

Most studies on GPA33 that are primarily focussed on imaging and/or as potential therapeutics in colorectal cancers were derived from a mouse monoclonal antibody that is often used as an scFV in its humanized form [39]. We recently reported on the construction of a novel scFV phage antibody library from companion animal cancer patients derived from cloning the naive B-cell repertoire [28]. An advantage of using this scFv naive library is that the animals have an ‘educated’ immune system being exposed to the same environmental conditions as their owners; i.e. these animals were not raised in pathogen-free animal houses. In addition, the advantage of in vitro screening on an antigen to isolate monoclonal antibodies is that the bait protein conformation in vitro can be controlled in a specific manner and maintained in a folded non-denatured state. By contrast, the immunization method, whereby an animal is injected with material mixed with an ‘adjuvant’, invariably denatures the antigen, potentially generating different epitopes than protein material presented using an in vitro screen with conformationally intact protein. Another advantage of the in vitro screen is that immunizing protein into animals usually requires hundreds of micrograms of antigen, but in vitro screening on immunoplate wells with phage libraries can use as little as tens of nanograms. This might be important for difficult-to-purify receptors for which the yield of folded, purified protein can be poor.

**Fig. 2:**
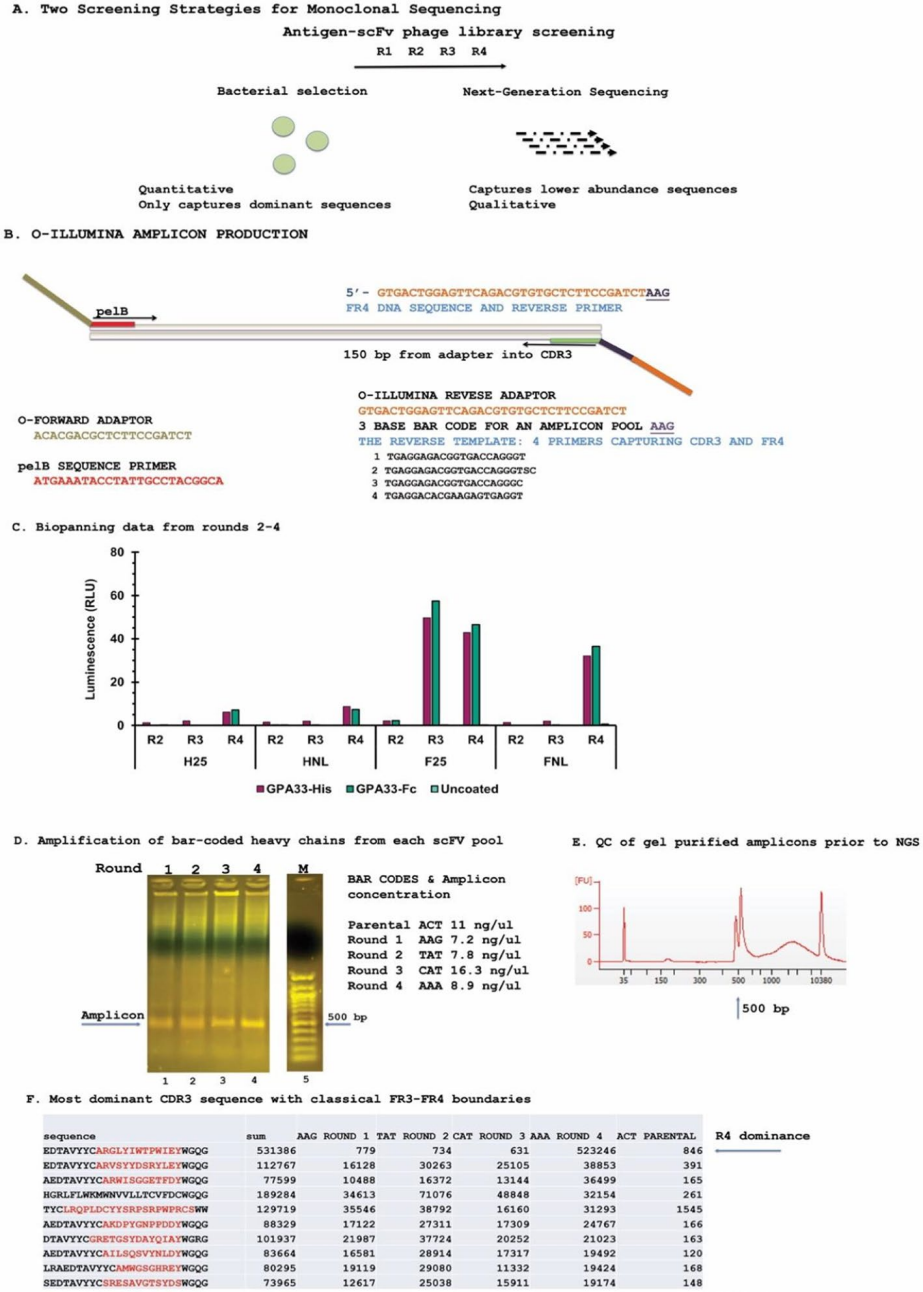
Next-generation short-read DNA sequencing of CDR3 amplicons. **A** Purified GPA33 was used as antigen in immunoplate ELISA wells and screened using four rounds of biopanning. These phage pools were validated using two independent assays: NGS CDR3 sequencing (this figure) and ELISA (Fig. 3). **B** After the final round of biopanning, each phage pool was used directly in PCR reactions with barcoded primers that aim to measure enriched CDR3 sequences as a function of rounds of biopanning [28]. **B** The position and sequence of the ‘old’ Illumina sequences (in brown, O-Illumina forward primer and, in orange, O-Illumina reverse barcoded primer), the 3-bp adapter position (in violet) and the sequences of the forward (pelB) and reverse primers (one forward primer and four reverse primers capturing FR4). **C** Isolation of monoclonal bacteriophage with anti-GPA33 activity using the pGEMP library. Following four rounds of biopanning, polyclonal bacteriophage pools were assayed for anti-GPA33 activity by ELISA. R2–R4 indicate biopanning rounds, pools named ‘F’ (FNL and F25) were raised against Fc-tagged GPA33. Those named ‘H’ (H25 and HNL) were raised against His-tagged GPA33. The labels ‘25’ and ‘NL’ refer to two different preparations of the pGEMP bacteriophage library from the master stock. The data are plotted as relative scFV phage binding (in relative light units (RLU)) as a function of the biopanning round and target. **D** The phage pools from **C** were processed using short-read NGS to measure the efficiency of biopanning. An example of the amplicons generated with these primers using phage pools R1–R4 from GPA33 screening with the ~ 500-bp amplicon highlighted. **E** Pooled amplicons were subjected to QC analysis using fluorometric methods involving the Invitrogen Qubit dsDBNA assay and quantified using an Agilent Bioanalyzer 2100 to define the amplicon molecular mass. The major amplicon of ~ 500 bp was quantified along with higher molecular mass DNA adducts that co-purified during gel electrophoresis. The amplicon was blunt end ligated to the new generation of Illumina adaptor primers and subjected to DNA sequencing by Source Bioscience (Cambridge, UK). The top 10,000 sequences were selected and analysed for DNA reads as a function of biopanning rounds with the parental library as the negative control (Supplementary Table 1 shows the top 10,000 sequences). **F** Examples of the ten most dominant antibody sequences enriched by round 4. The most highly enriched CDR3 domain isolated appeared in round 4 with over 500,000 sequence reads after PCR and NGS. Additional clones with different CDR3 sequences that enriched in round 1 but did not over-dominate by round 4 are also shown. The data are presented as the number of reads as a function of each round of biopanning and include the number of reads detected in the parental library. The data do not directly reflect the actual abundance of any one antibody sequence because data generation required over 30 cycles of PCR to generate the amplicons for NGS sequencing, which in turn required additional amplification stages. Thus, the relative read number provided a qualitative gauge of abundance with the caveat that there would be a relatively low sequence bias affecting amplicon production during the PCR cycles

**Fig. 3:**
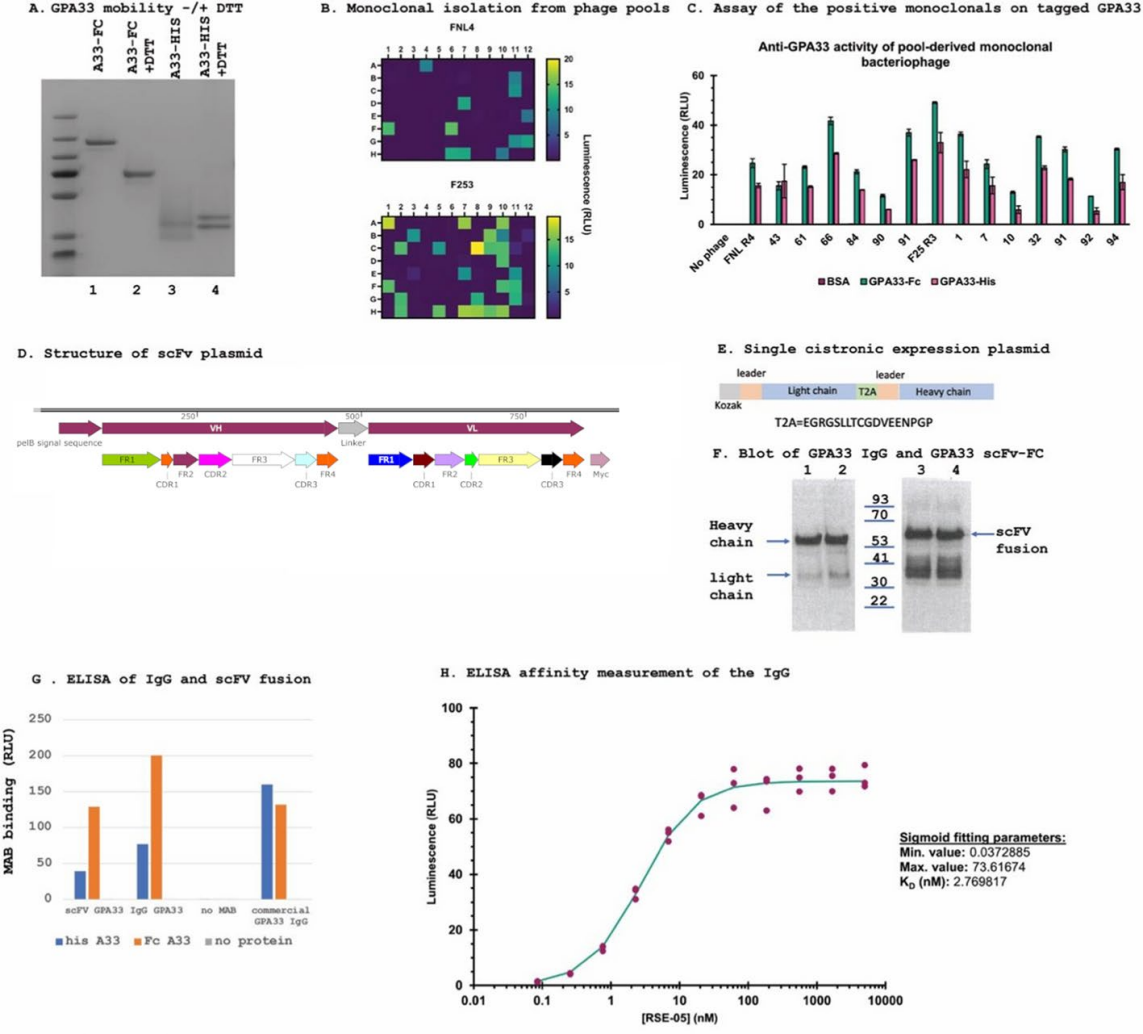
Isolation of a GPA33-specific antibody from a scFV phage display library. **A** Relative purity of GPA33 proteins used as bait in antibody screening. SDS gel electrophoresis was used to separate FC-tagged GPA33 and His-tagged GPA33 without and with DTT. The gel was stained with Coomassie Blue; lanes are markers, on the far left; lane 1, Fc-GPA33; lane 2, Fc-GPA33 + DTT; lane 3, His-GPA33; lane 4, His-GPA33 + DTT. **B** Monoclonal bacteriophage from phage pools (Fig. 2C) were isolated and propagated by picking single colonies of ER2738 infected with bacteriophage from agar plates, and tested for anti-GPA33 activity by high-throughput ELISA. FNL4 refers to round 4 phage from the FNL screen using FC-tagged GPA33, and F253 screen refers to the F25 screen from round 3 (as in Fig. 2C). **C** Numbered monoclonal scFvs from two biopanning pools (labelled FNL4 and F253, **B** and Fig. 2C) were surveyed for binding activity versus recombinant GPA33, from two different sources (His and Fc tagged). BSA was used as a negative control for antigen-binding activity. **D** Sequence structure of the synthetic scFV. The sequence of CDR3 from all active phage scFV monoclonals from round 4 matched the dominant sequence in Fig. 2F (top row) with 523,246 sequencing reads. **E**, **F** Production in ExpiCHO cells and ELISA. A plasmid with a single cistronic mRNA encoding light and heavy chains of this abundant active clone, separated by the T2A translation termination sequence (**E**), was used to produce (**F**) either an immunoblot developed with ECL showing the heavy and light chain from an IgG encoding plasmid (lanes 1 and 2) or scfv-Fc fusion protein encoding plasmid (lanes 3 and 4). **G** Activity of the RSE-05 antibody as either a scFv-FC fusion or IgG. ELISA was used to measure the activity of the ExpiCHO-produced antibodies. Fc-GPA33 or his-GPA33 were coated onto immunoplates, and binding was measured using ECL. **H** ELISA was used to estimate the affinity of RSE-05 for the extracellular domain of GPA33 bound to a solid phase. Binding of RSE-05 to GPA33 was measured across a concentration range of RSE-05, to determine the concentration of RSE-05 which gives 50% of maximum binding, and thereby an estimate of dissociation constant. Triplicate measurements are in purple, with fitting performed as described [67]

We aimed to screen the scFv library against conformationally intact GPA33 protein using small volumes in immunoplate wells and then use both short-read NGS methodologies and colony isolation to identify enriched monoclonal antibodies (Fig. 2A, B). The integration of NGS for quantifying the extent of CDR3 enrichment, to the classic biopanning process, is a recently evolved methodological step that aims to measure and document the efficacy of biopanning [28]. Four rounds of biopanning against two eukaryotically expressed GPA33 were performed, His- and Fc-tagged (see Fig. 3A for relative protein purity). Biopanning against His-tagged GPA33 yielded weak enrichment of anti-GPA33 activity (Fig. 2C, panels ‘H25’ and ‘HNL’), whereas Fc-tagged GPA33 proved a more effective biopanning substrate (Fig. 2C, panels ‘F25’ and ‘FNL’). Those named ‘H’ (H25 and HNL) were raised against His-tagged GPA33, and pools named ‘F’ (FNL and F25) were raised against Fc-tagged GPA33. The numbers ‘25’ and ‘NL’ refer to two different preparations of the pGEMP bacteriophage library from the master stock.

Despite the poor enrichment of antibody pools against GPA33-His, the GPA33-Fc derived scFV-bacteriophage showed roughly equal binding to each of the GPA33 formulations (Fig. 3C, ‘F25’ and ‘FNL’ panels). These data indicate that the epitope(s) targeted by these scFV-bacteriophage are intact in the GPA33-His protein. Figure 3A shows that the GPA33-His preparation presents two distinct species by SDS-PAGE, and in the non-denaturing condition these species form a smear. This heterogeneity is presumed to contribute to the poor enrichment of using the His-tagged antigen by biopanning, despite the existence of the same epitope(s). This example emphasizes the importance of antigen quality, or conformational state, for successful biopanning.

After the final rounds of biopanning (Fig. 2C), the phage pools were used in PCR reactions with barcoded primers (Fig. 2B) that aim to measure enriched CDR3 sequences as a function of rounds of biopanning [28]. Pooled amplicons from all four rounds (Fig. 2D) were subjected to QC analysis prior to blunt ended ligation of the Illumina sequencing adaptors (Fig. 2E). The amplicon was blunt end ligated to the new generation of Illumina adaptor primers and subjected to DNA sequencing. Sequencing reads containing the indicated barcodes (Fig. 2D) were extracted from the raw sequencing files (data not shown). The top 10,000 enriched sequences from rounds 1–4 and including the parental library are shown in Supplementary Table 1. The top 10 antibody sequences from this list identified are shown in Fig. 2F, with the most highly enriched CDR3 domain isolated (top row) appearing in round 4 with 523,246 sequences (Fig. 2F). The other CDR3 sequences exhibited a ~ 10× lower representation in round 4 PCR amplified DNA reads versus the most abundant CDR3 sequence. This might be due to the lower concentration of these antibody clones in the starting parental library. In addition, several of these other sequences were enriched beginning in round 1 and did not increase in abundance through round 4 (Fig. 2F). We next asked the question of to what extent the enriched sequences from NGS data (Fig. 2F) overlap with clones chosen by traditional colony isolation and Sanger sequencing (Fig. 3).

The phage pools subjected to four rounds of biopanning against GPA33 were also independently validated using monoclonal colony isolation and ELISA (Fig. 3). Two active phage pools (FNL-4 and F25-3, representing round 4 and round 3, respectively, from Fig. 2C) were plated to isolate individual monoclonal antibody expressing phagemids. Media from 96 of each of ampicillin (AMP)-resistant colonies were tested for GPA33 binding by ELISA (Fig. 3B). The active antibodies from this ELISA were retested against His- and Fc-tagged GPA33; representative monoclonal scFV phage that bind to both GPA33-Fc or His-tagged GPA33, but not BSA, are highlighted in Fig. 3C. Upon Sanger sequencing of several of these monoclonal antibodies, the sequences were all identical (data not shown). The CDR3s sequenced from individual colonies matched the single abundant clone identified using NGS from Fig. 2F (top row with 523,246 sequencing reads), suggesting that the most abundant antibody isolated using colony screening is a direct function of the abundance in the phage pool as detected by NGS. It would be interesting to characterize the other, less abundant antibody sequences enriched in round 4 (Fig. 2F and Supplementary Table 1), but this is beyond the remit of this study. These include sequences such as SRESAVGTSYDS (Fig. 2F, last row), where 148 sequences were identified in the parental library and 19,174 sequences were identified in round 4. This represents 3.6% of the enriched sequences, and we would consider this part of the rarer group of phage sequences whose isolation would require the design of PCR primers, on the basis of the DNA sequence, to clone out the heavy and light chains associated with this antibody gene.

The aim of this study was not to isolate as many distinct GPA33 antibodies as possible but to identify a lead antibody that could be used for validation as a diagnostic tool. The gene structure of a representative scFV is shown in Fig. 3D, and we named this antibody RSE-05. Features include the *pelB* leader sequence, the VH gene linked to the VL gene and containing a C-terminal MYC tag. To test the bioactivity of the RSE-05-scFv, it was incorporated into an IgG and an scFV-FC fusion protein expression plasmid using gene synthesis. This is important as scFv sequences cannot always be converted into an IgG, as reported recently for scFv targeted to PD-1 [40]. In both IgG and an scFV-FC gene fusions, mouse IgG2b Fc sequences were used because of availability of robust secondary reagents targeting mouse constant domains.

The RSE-05 MAB gene was synthesized as an IgG using one cistron containing an ER leader sequence prior to the light-chain and heavy-chain genes and fused a ‘self-cleavage’ sequence derived from virus T2A, which theoretically allows the production of equal mixtures of heavy- and light-chain gene from one plasmid (Fig. 3E). When this plasmid was transfected into ExpiCHO cells and media was collected after 10 or 12 days, immunoblotting revealed that heavy- and light-chain proteins were produced (Fig. 3F, lanes 1 and 2). The RSE-05 scFV-FC fusion protein also contained an ER leader sequence, and significant amounts were detected in the media of ExpiCHO cells of the correct mass (Fig. 3F, lanes 3 and 4), which is slightly larger than the heavy chain from the IgG (~ 50 kDa). The purified scFV-Fc fusion also contains lower-molecular-weight fragments that could be translation termination or degradation products. Initial analysis indicated that both the RSE-05 IgG and the RSE-05 scFV-FC fusion protein were active in an ELISA towards His-tagged and Fc-tagged GPA33 protein (Fig. 3G). These data are consistent with the scFv-phage pools and indicate, firstly, that the RSE-05 scFv can be constructed into an IgG scaffold and still retain activity when produced in a mammalian cell. In addition, the RSE-05 scFV-FC itself retains activity as an orthogonal fusion protein (FC rather than gIII protein), again suggesting that it can be manipulated into different synthetic structures in the future.

Finally, we estimated the relative affinity of the Expi-CHO produced IgG for the antigen. ELISA has been previously reviewed as a method to complement surface plasmon resonance (SPR) in the generation of a *K*_d_ for an antibody–antigen complex [41, 42]. Using a derivation of the ELISA method that titrates IgG concentration with a fixed concentration of antigen on the solid phase, the *K*_d_ of the RSE-05 monoclonal antibody for GPA33 bound to solid phase was estimated to be ~ 3 nM by ELISA (Fig. 3H). As we anticipate the use of RSE-05 as a future diagnostic lead antibody, we are currently subjecting the antibody to light-chain shuffling and additional targeted mutagenesis in attempt to increase the affinity, where on–off rates would be measured by SPR rather than by ELISA.

### Binding of the RSE-05 MAB to the outer membrane of living cells

As stated above, we used eukaryotically expressed GPA33, rather than bacterial produced GPA33 or peptide antigens derived from GPA33, to screen for monoclonal antibodies to increase the probability of acquiring antibodies that bind to the native receptor. Although RSE-05 binds to this recombinant protein, it was important to determine whether the antibody bound to GPA33 when the protein was assembled into the membrane of living cells within proteo-lipid rafts.

**Fig. 4:**
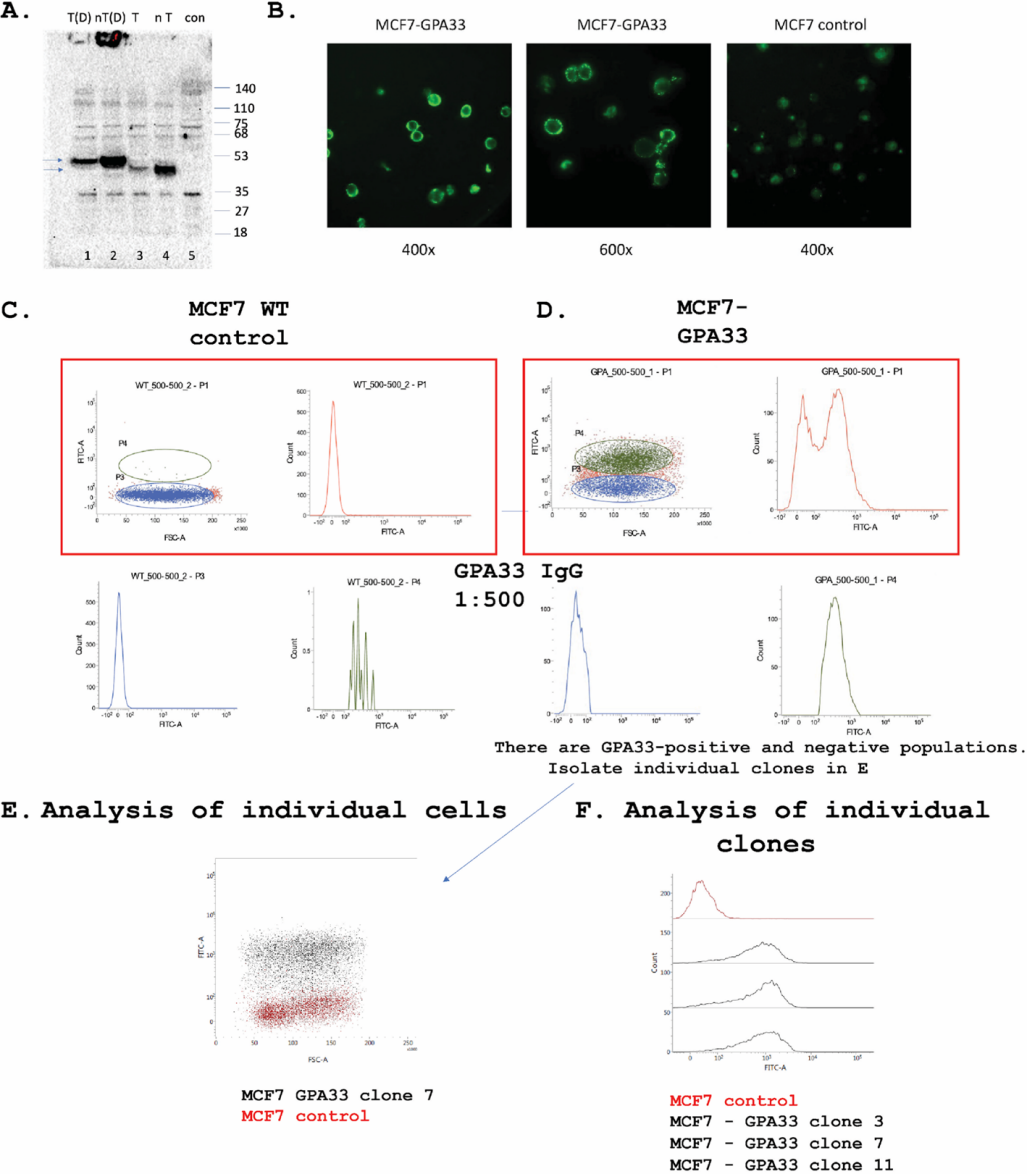
The RSE-05 MAB binds to full-length GPA33 protein after transfection into human cells. **A** Validation of GPA33 expression plasmids in human cells. MCF7 cells were transfected with either HA-tagged (T) (lanes 1 and 3) or non-tagged (nT) full length GPA33 containing the transmembrane domain (lanes 2 and 4) expression plasmids, and after acquiring the cell lysates using urea lysis buffer (8 M urea in PBS), a denaturing immunoblot was processed in lanes that either contain DTT (D) (lanes 1 and 2) or without DTT in the sample loading buffer (lanes 3–5). Lysates from non-transfected MCF7 cells are in lane 5. Samples were immunoblotted with the Atlas pAb (commercial antibody), which can detect all isoforms of GPA33. The presumed monomeric form of GPA33 has a faster mobility in the absence of DTT, which presumably reflects SDS resistance of the two IgG-like lobes to denaturation due to the three di-sulphide bonds. **B** Analysis of RSE-05 MAB binding to GPA33 in fixed cells using immunofluorescence. **C**, **D** Analysis of RSE-05 MAB binding to cell surface localized GPA33 in living cells. MCF7 cells were mock transfected (**C**) or transfected with the GPA33 expression plasmid (**D**) and processed by flow cytometry. The data are plotted as: left panels, FITC-A is plotted as a function of FSC-A; right panels, cell count is plotted as a function of FITC-A (left panels and right panels). **E** MCF7 cells were stably transfected with the GPA33 expression plasmid and flow cytometry was used to isolate the positive cells (in grey) separated from the GPA33 negative cells (in red). The data are plotted as: FITC-A as a function of FSC-A stable GPA33-positive cells and GPA33-negative negative cells. **F** Analysis of individual GPA33 + cell clones, 3, 7 and 11. Three GPA33 stably expressing cell clones were processed by flow cytometry to measure GPA33-positive staining with the RSE-05 MAB. The data are plotted as: GPA33-positive cells as a function of FITC-A

Transfection of the full-length GPA33 gene into MCF7 cells can result in the expression of the protein at the approximate molecular mass using the commercial rabbit polyclonal antibody to GPA33 (Fig. 4A). The data show that both untagged (nT) and HA-tagged GPA33 can be expressed in MCF7 cells (Fig. 4A, lanes 3 and 4). In addition, the inclusion of DTT in the SDS loading buffer also reduced the protein as evidenced by the increase in molecular mass in the SDS denaturing gel (Fig. 4A, lanes 1 and 2). However, the protein expressed from the full-length GPA33 gene (containing the transmembrane domain and intracellular peptide motifs) in MCF7 cells produces only one protein band, whilst the recombinant protein containing the extracellular domain only, purified from the supernatant of HEK293 cells, produces two bands (Fig. 3A, lane 4 versus 3).

Having shown that the GPA33 expression vectors can produce full-length GPA33 protein in HEK293 human cells, we also tested whether we could use OAC cell lines to measure endogenous GPA33 in living cells. However, none of the five commonly available OAC cell lines (FLO-1, OE33, OE19, JH-Eso-Ad1 and OAC4PC) produce endogenous GPA33 protein (Supplementary Fig. 4). Four of the five OAC cell lines (FLO-1, OE33, JH-Eso-Ad1 and OAC4PC) can be seen to produce the GPA33 protein after GPA33 plasmid transfection (Supplementary Fig. 4A–D). We thus determined whether the epitope of the antibody is accessible on living cancer cells; for example, it is possible that the receptor would be in a modified state or have an altered epitope on the cell surface that precludes the antibody binding to its epitope.

Adherent cells were transfected again with the GPA33 expression plasmid, and then living cells attached to the plate were incubated with the RSE-05 MAB followed by incubation with a fluorescently tagged secondary antibody and visualized by immunofluorescence (Fig. 4B). The RSE-05 MAB can bind to the outer surface of non-permeabilized adherent MCF7 cells versus the control non-transfected cells (Fig. 4B). When the transfected adherent cells are dissociated by trypsin into living cell suspensions, the non-permeabilized full-length GPA33 transfected cells also can bind the RSE-05 MAB (Fig. 4C–F).

**Fig. 5:**
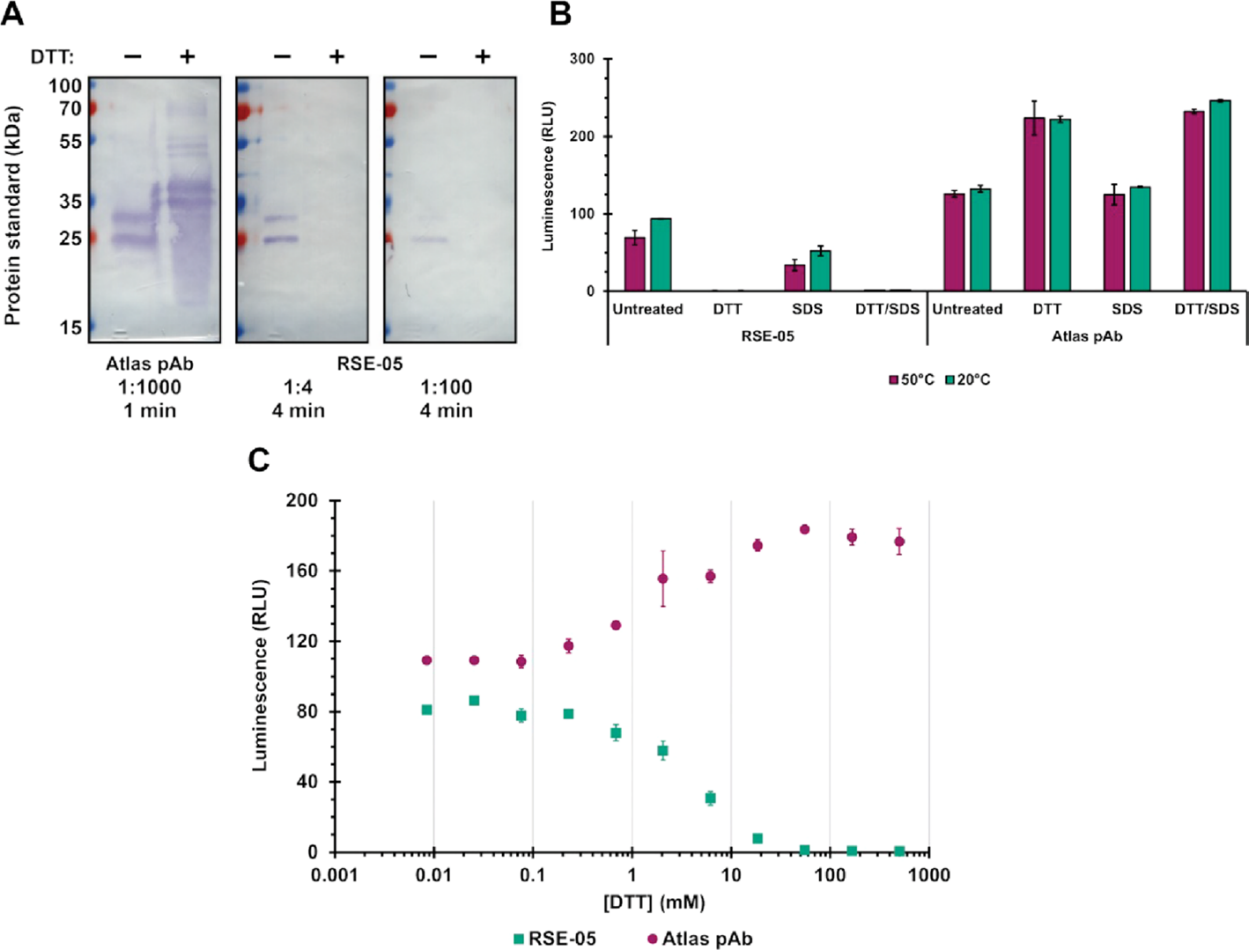
The epitope of the RSE-05 IgG is sensitive to reduction. **A** The indicated antibodies were used in immunoblots using purified His-tagged GPA33 protein without or with DTT in the SDS loading buffer. After incubating with the primary antibodies and adding anti-mouse or anti-rabbit HRP-conjugated secondary antibodies, membranes were stained using TMB. **B** ELISA was used to measure the binding activity of the RSE-05 MAB compared with the commercially available rabbit polyclonal antibody (Atlas pAb). Using GPA33 treated with the indicated chemicals (0.5% v/v SDS and/or 50 mM DTT), at different temperatures, RSE-05 and Atlas pAb (commercial antibody) binding was evaluated using anti-mouse or anti-rabbit HRP-conjugated secondary antibody, respectively. The binding activity is measured in luminescence, relative light units. **C** ELISA performed as in **B**, in this case with DTT titrated

**Fig. 6:**
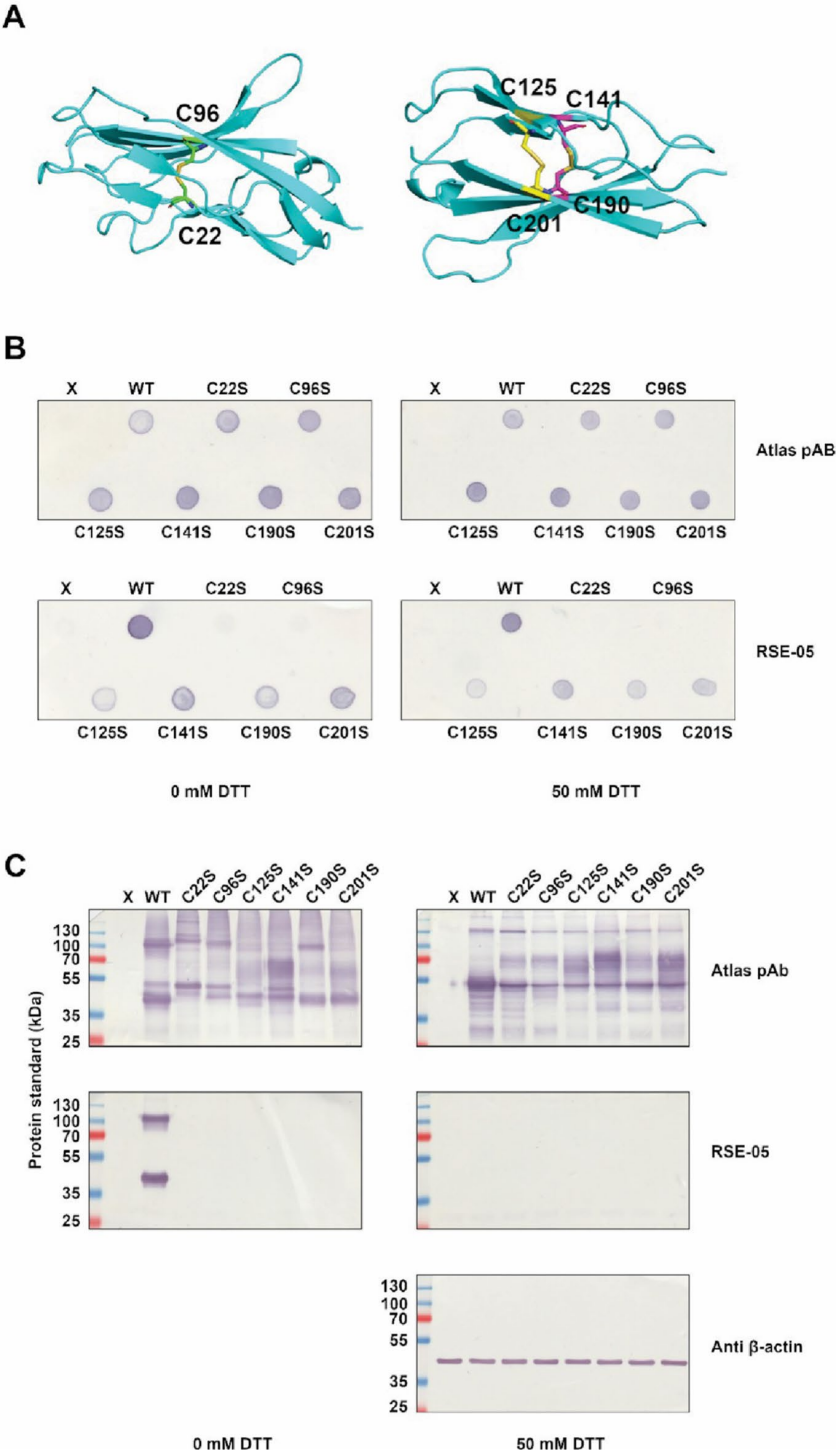
Characterization of redox-sensitive epitope using cysteine mutants. **A** The di-sulphide bonds of GPA33 (green, yellow and magenta) indicated on a ColabFold [44]-generated GPA33 structure (cyan). The Ig-like V type (left) and Ig-like C2-type (right) are shown separately. **B** Immuno-dot-blots of HEK293T cell lysates following transfection with wild-type GPA33/cysteine-mutant GPA33 expression vectors. Lysates were first reduced with 50 mM or 0 mM DTT. Sample ‘X’ is untransfected control. Membranes shown are representative from three biological replicates. **C** Denaturing SDS-PAGE immunoblots of HEK293T cell lysates. As in **B**, lysate was treated with 50 mM or 0 mM DTT prior to separation by SDS-PAGE

It remains possible that the GPA33 isoforms we use (HEK293 cell-expressed extracellular receptor domain used in biopanning and the transfected full-length protein in cancer cells) do not recapitulate the receptor conformation within human cancer tissue in vivo. Analysing the antibody using fresh surgically resected, living cancer tissue is not within the remit of our current study. Nevertheless, the ability of the antibody to function in the setting of flow cytometry of GPA33-transfected cancer cells is a proof of concept that the antibody can bind not only to the recombinant, HEK293 cell-expressed extracellular receptor domain but also to full-length protein containing its transmembrane domain, in living cells. These data support future testing of the RSE-05 antibody on freshly resected, living clinical tissue. In addition, there are similarities between our two model systems; both the HEK293 expressed extracellular domain of GPA33 and the transfected full-length GPA33 have epitopes that are both sensitive to DTT (Fig. 5 and 6, respectively). In addition, XL-MS experiments used HEK293 expressed extracellular domain of GPA33 to map cross-links sites in GPA33 lobe 1 (Fig. 8), which is consistent with the mutagenesis data using transfected full-length GPA33 (Fig. 6). Altogether, we can conclude that the HEK293-expressed GPA33 receptor domain, and the transfected full-length GPA33 protein, ‘folds’ in a similar manner, with respect to the di-sulphide bond architecture that constrains the RSE-05 monoclonal antibody epitope.

### The RSE-05 MAB epitope requires an oxidized protein and is sensitive to reducing agent

The ability of RSE-05 MAB to bind the receptor in living cells prompted us to understand more about the nature of the epitope because it is of interest to develop the antibody for use in diagnostic imaging. Because receptors often have di-sulphide bonds that are highly regulated by the ER chaperone system, we first evaluated whether the RSE-05 MAB epitope is sensitive to changes in the redox state. For example, daratumumab binds to an epitope on CD38 that is abrogated by the treatment of cells with DTT [43].

First, we performed an immunoblot of His-tagged GPA33 protein with and without reducing agent; the commercial antibody could be used to demonstrate that the His-tagged GPA33 protein epitope is not lost upon DTT treatment using the commercial antibody (i.e. ‘Atlas pAb’, Fig. 5A, left panel), although the migration of the protein in a denaturing gel is faster without DTT (Fig. 5A, left panel). By contrast, RSE-05 MAB was able to immunoblot denatured his-tagged GPA33 protein but not reduced and denatured GPA33 protein (Fig. 5A, middle and right panels).

This was also evaluated using ELISA. With His-tagged GPA33, the RSE-05 MAB remained active by ELISA (Fig. 5B, untreated). However, unexpectedly, the use of either DTT or SDS/DTT preincubation of GPA33 protein abrogated IgG binding (Fig. 5B, DTT or DTT/SDS), whilst incubation of GPA33 with SDS alone had no effect. As a control, the commercially available GPA33 rabbit polyclonal antibody was active in binding to His-tagged GPA33 protein (Fig. 5B, ‘Atlas pAb’ panel) and was stimulated marginally by the pre-inclusion of DTT or DTT/SDS into the GPA33 protein solution. These data suggest that the antibody epitope is conformationally sensitive and presumably requires Cys–Cys bonds for the epitope to be recognized.

Titration of DTT in ELISA demonstrated the concentration at which the GPA33 protein loses the RSE-05 MAB epitope, which is approximately 10 μM under the experimental conditions we employed (Fig. 5C). By contrast, the commercial pAb exhibits a mirror increase in binding to GPA33 protein upon titration of DTT, suggesting that the pAb epitope is partially constrained by oxidation (Fig. 5C).

### Probing the dominant redox-regulated RSE-05 MAB epitope using cysteine mutated isoforms of GPA33

GPA33 is composed of two IgG-like domains; lobe 1 has a V-type IgG domain, and lobe 2 has a C2-like IgG domain (Fig. 6A, generated using ColabFold [44]). The Ig-like V type domain consists of two beta-strand dominated folds, bridged by a single di-sulphide bond. The Ig-like C2 type domain consists of two well-defined beta-sheets, bridged by two di-sulphide bonds. There are three potential di-sulphide bonds which might comprise the epitope (Fig. 6A, C22–C96, C125–C201 and C141–C190.)

All six di-sulphide bond-forming cysteines in GPA33 were converted to serine and expressed in human cells to determine whether any single missense mutation abrogated the RSE-05 MAB epitope. Using a dot blot, which is thought to retain some non-denatured conformational character, mutation of cysteine 22 or 96 to serine destroyed the RSE-05 MAB epitope (Fig. 6B, ‘RSE-05 MAB’ panel versus ‘Atlas pAb’ panel). The other cysteine to serine mutant forms of GPA33 did not disrupt the epitope to equivalent levels (Fig. 6B, ‘RSE-05 MAB’ panel versus ‘Atlas pAb’ panel). Notably, only loss of the C22–C96 bond disrupted the epitope in the dot-blot, but all six of the C–S mutations abrogated binding in SDS-PAGE immunoblot, even in the absence of reduction (Fig. 6C, 0 mM DTT blots, ‘RSE-05 MAB’ panel versus ‘Atlas pAb’ panel). The difference in binding between Fig. 6B and C indicates that either the addition of SDS or the heating of the sample, when in combination with any of the six C–S mutations, is sufficient to disrupt the RSE-05 epitope. Moreover, the ability of all six C–S mutations to disrupt RSE-05 binding in a denaturing immunoblot indicates that the two domains are not structurally independent, and the RSE-05 epitope may be sensitive to this. These data suggest that the primary RSE-05 MAB epitope for GPA33 resides in lobe 1, the V-type IgG domain (Fig. 6A), and that the epitope is highly sensitive to tertiary structure disruption in lobe 2, the C2-like IgG domain. The inclusion of DTT in the loading buffer changes the mobility of the ‘monomer’ and ‘dimer’ of GPA33 (Fig. 6C, 50 mM DTT top blot, ‘Atlas pAb’ panel). The mobility of all the cysteine mutants is altered and are different from the wild-type mobility and from each other (Fig. 6C, 50 mM DTT top blot, ‘Atlas pAb’ panel), suggesting that the unfolding of the two lobed domain GPA33 protein is sensitive to local conformational perturbation caused by a single cysteine mutation. The inclusion of DTT in the sample buffer partly normalized the mobility so that all mutant forms of GPA33 share the mobility of the monomeric wild-type GPA33 and the high-molecular-mass species. In addition, all cysteine mutants exhibit a smear of intermediate mobility (Fig. 6C, 50 mM DTT top blot, ‘Atlas pAb’ panel), further suggesting that the folding of the mutant proteins in an SDS gel exhibits allosteric effects.

**Fig. 7:**
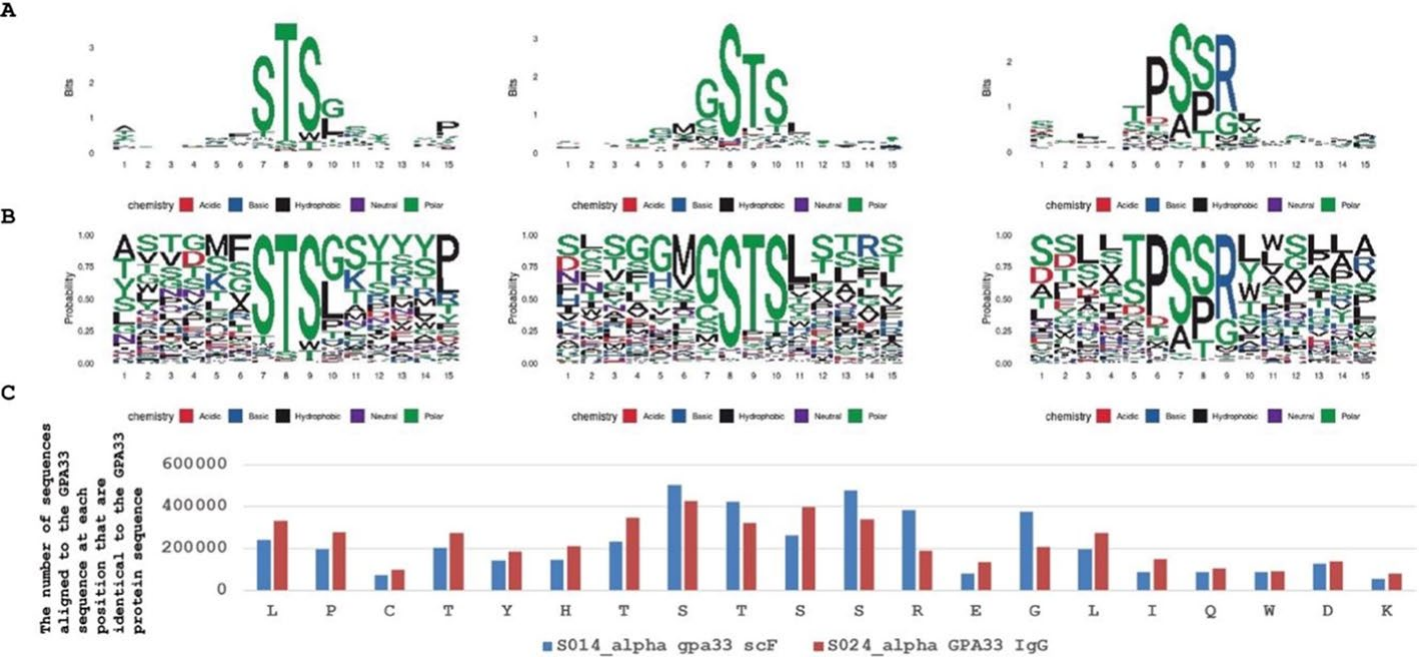
The anti-GPA33 IgG RSE-05 and scFv-FC fusion antibody was purified from ExpiCHO cells and as bait to define a potential linear epitope component using peptide phage display. Purified RSE-05 IgG or the scFv-FC fusion was captured on protein A beads and incubated with the peptide-phage library, followed by one round of biopanning and next-generation sequencing. The sequencing data was used to derive images from a de novo analysis using Hammock software [30]. **A**, **B** The three presented images are derived from Hammock software as the three best motifs, left to right. The web logo (relative amino acid frequencies) shows the most enriched sequence clusters for the GPA33 antibodies. **A**, **B** The size of each amino acid corresponds to its relative frequency at a given position within the cluster. **A** Bits are plotted a function of amino acid at each position to measure how much information (in bits) a given position carries and how ‘certain’ or ‘variable’ this information is. The data are expressed in terms of Shannon information as reported previously [28]. **B** Probability plotted as a function of amino acid at each position, which indicates the relative frequency (probability of occurrence) of a particular amino acid at a given position in a multiple sequence alignment (MSA) as reported previously [28]. **C** The graph shows the number of sequences (*y*-axis) aligned to the amino acid sequence of the GPA33 protein (*x*-axis). The *x*-axis represents the GPA33 protein sequence (aa 41–60), and the *y*-axis represents the number of sequences aligned to the GPA33 sequence at each position that are identical to the GPA33 protein sequence. The RSE-05 IgG binding results are plotted with red bars (S024), and the RSE-05 scFv-FC fusion binding data are plotted with blue bars (S014)

The RSE-05 IgG and the scFV-FC fusion antibody were next subjected to peptide phage display using a 12-mer combinatorial peptide library (Fig. 7) and then extracted into motifs as previously reported for peptide phage display library use after one round of biopanning [30]. The three images (Fig. 7A, B, left to right) summarize the web logo in the form of relative amino acid frequencies of the most enriched sequence clusters for the GPA33 antibodies and the size of each amino acid corresponds to its relative frequency at a given position within the cluster. An additional way to plot the data is to plot the number of sequences as a function of the amino acid sequence of the GPA33 protein (Fig. 7C). The polypeptide sequences that emerged identified amino acid stretches enriched with a core ‘STS’ motif. This peptide motif resides adjacent to cysteine 22 as positions 27–29 in the sequence 22-CTYHTSTSSREGLIQW-37. These data suggest that the primary epitope of RSE-05 resides within lobe 1 of GPA33 protein and that it is possible the epitope is stabilized by the Cys22–Cys96 di-sulphide bond.

### Cross-linking mass spectrometry (XL-MS) identifies an interface in the IgG–antigen complex

**Fig. 8:**
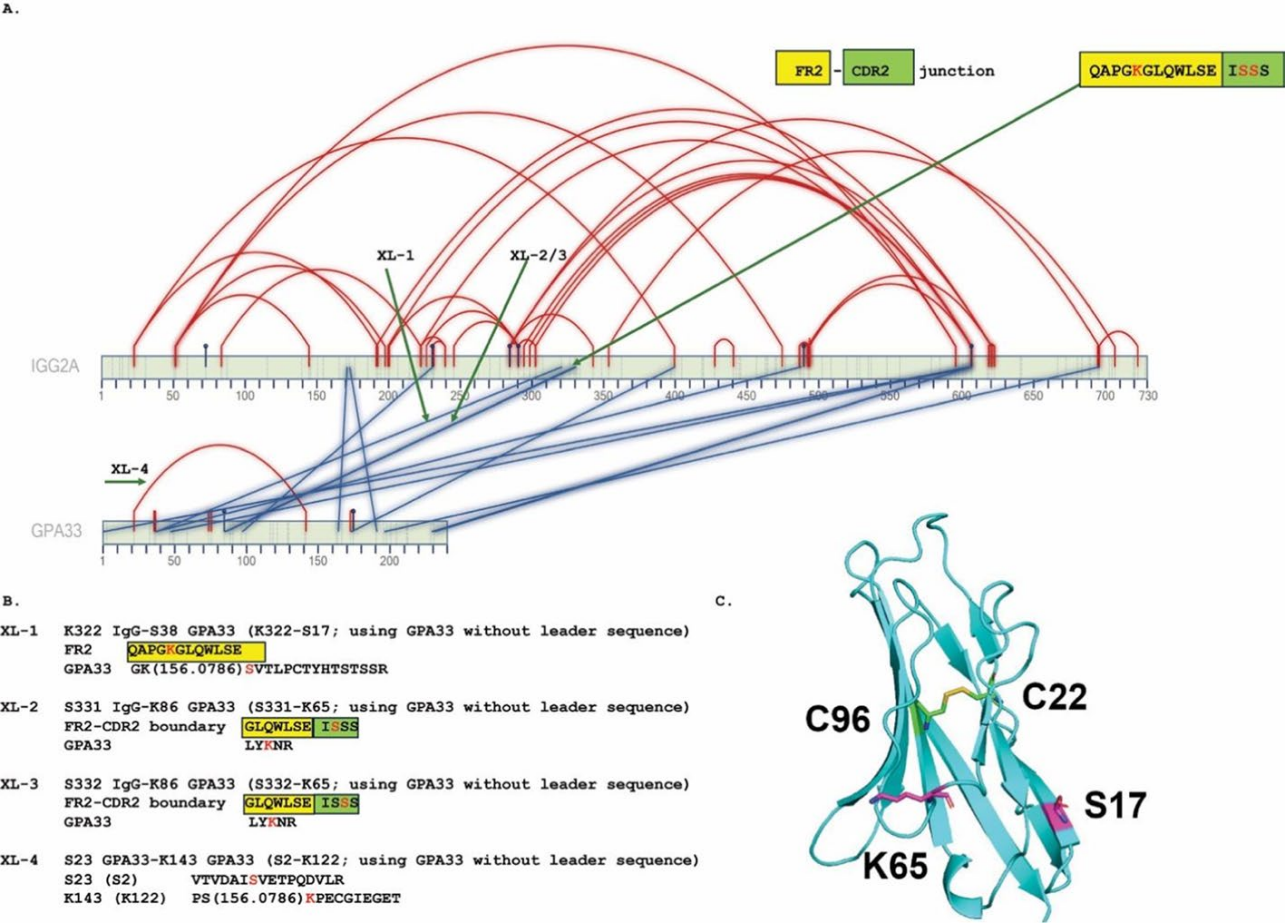
XL-MS identifies sites of interaction between CDR2-FR2 of the RSE-05 IgG and lobe 1 of GPA33. **A**. Overall analysis of the cross-linked adducts between the IgG and antigen identified using XL-MS [32]. **B** Detailed sequence analysis of the peptide adducts identified containing the DSS cross link. XL-1, 2, 3 and 4 are marked using green arrows. Yellow highlights FR2 sequences, and green highlights CDR2 sequences. **C** Position of Ser17 and Lys65 (in magenta colour) cross-linked amino acid sites on GPA33 lobe 1 in relation to the Cys22–Cys96 di-sulphide bond (in yellow)

We next subjected RSE-05 IgG and GPA33 to chemical cross-linking mass spectrometry to localize the epitope, as previously reported [32]. The GPA33 protein and the IgG were mixed and subjected to covalent cross-linking (Supplementary Fig. 5). Both EGS and DSS were able to show titratable high-molecular-mass species (Supplementary Fig. 5), and as such, the reactions were scaled up using 5 mM of each cross-linking agent for gel trypsinization. Following deconvolution of the DSS cross-linked mass spectra [32] (Fig. 8A), covalent adducts were identified between amino acids in the CDR2 and CDR2–FR2 interface of the IgG and two discontinuous amino acids in GPA33 V-type domain at amino acids Ser17 and Lys65 within lobe 1 (Fig. 8B). Ser17 and Lys65 are projected to one ‘face’ of the GPA33 protein when the structure is constrained by the Cys22–Cys96 di-sulphide bonds (Fig. 8C). This provides further evidence suggesting that RSE-05 IgG binds primarily to lobe 1 of GPA33 protein, which is constrained by the Cys22–Cys96 di-sulphide bond.

### The adaptability of RSE-05 in a two-site sandwich ELISA format

To evaluate the versatility of RSE-05 as a potential diagnostic reagent in a two-site capture (solid phase) or sensor (liquid phase) ELISA format, we needed to access its activity in the capture or sensor format using antibodies that presumably do not bind to the same regions. For example, it is possible, owing to the complex nature of the RSE-05 epitope, that no available antibodies can function in combination as a diagnostic pairing with RSE-05. This would eliminate RSE-05 as a possible future diagnostic tool in this format.

**Fig. 9:**
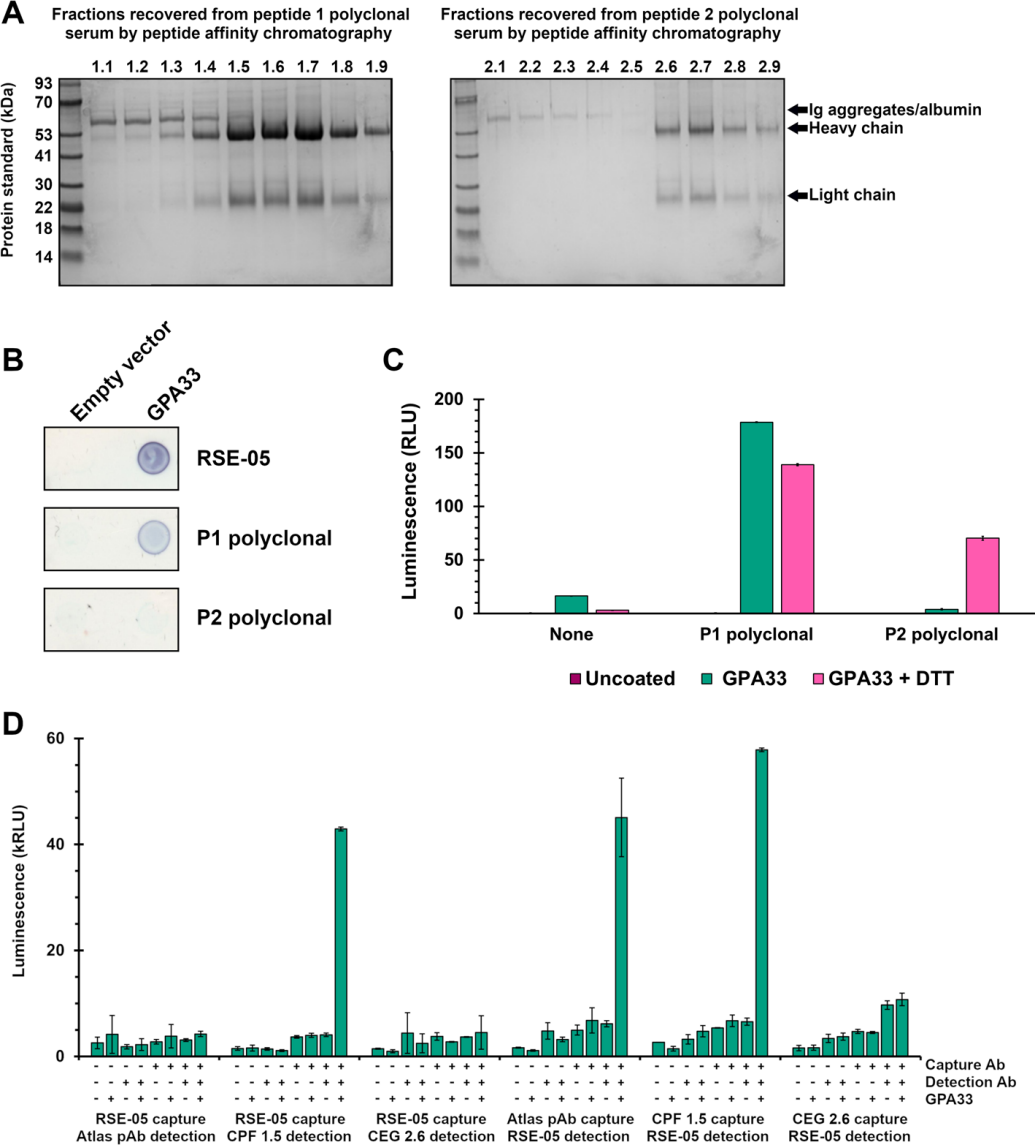
Development of antibodies to detect captured GPA33 in an ELISA format. Evaluation of RSE-05 in capture-sensor ELISA format using three distinct polyclonal antibodies to GPA33. **A** The indicated sera (from peptide 1 and peptide 2 derived from potential GPA33 surface loops; Supplementary Fig. 6) were validated for presence of peptide specific IgG; the sera was affinity purified on a peptide 1 or peptide 2 column, respectively, and after elution with 0.1 M glycine buffer (pH 2.5), fractions were reduced and separated on an SDS gel and stained with Coomassie Blue to validate the presence and amounts of peptide-specific IgG. **B** Dot-blotting of transfected HEK293T cell lysates without or with GPA33. 7.5 μg of protein lysate was used in each dot. Left, empty-vector transfections. Right, GPA33-gene transfections. Polyclonal fractions 1.5 and 2.6 were used for peptide 1 and peptide 2 respectively. **C** Anti-GPA33 activity of polyclonal sera by ELISA format, fractions 1.5 and 2.6. 200 ng of tagged GPA33 was coated, or with pre-treatment using 50 mM DTT to reduce di-sulphide bonds, with uncoated wells as negative control. **D** Sandwich ELISAs using three different polyclonal antibodies in conjunction with RSE-05 to determine whether RSE-05 can function in a capture-sensor format. The indicated antibodies were coated to the immunoplate wells as capture agents (RSE-05, Atlas polyclonal antibody, CPF1.5 P1 polyclonal antibody or CEG 2.6 P2 polyclonal antibody). Following this, 200 ng of recombinant GPA33 protein (FC tagged) was added then complementary antibodies were added in sensor format, labelled as ‘detection’. Anti-mouse or anti-rabbit HRP secondary antibodies were then added, and signal measured using HRP and luminescence

To address the adaptability of RSE-05, we used a commercial antibody to GPA33 (with unknown, proprietary epitopes, thus we do not know if they overlap with the RSE-05 epitope) and we generated two different polyclonal sera to peptides to distinct surface domains in GPA33 to minimize the likelihood of steric hindrance towards RSE-05 (Supplementary Fig. 6). The theoretical structure of GPA33, defined using AlphaFold, was used to identify two surface regions that might be compatible as capture or sensors (Supplementary Fig. 6). Two peptides derived from these surface domains were immunized into rabbits, and the polyclonal sera was affinity purified on peptide columns to determine the amount of specific IgG acquired (Fig. 9A). Peptide 1 (P1) yielded more polyclonal antibody than peptide 2 (P2); nevertheless, both antigens gave rise to bioactive polyclonal antibodies after the P1 or P2 affinity purifications.

The peak SDS gel fractions of P1 or P2 polyclonal antibodies were tested using lysates from cells transfected with full-length GPA33 in a dot blot (Fig. 9B), to measure whether the antibodies can bind to GPA33 containing its transmembrane domain. Both antibodies function in the crude lysate from cells expressing GPA33, although the P2 polyclonal antibody was much weaker than the P1 polyclonal antibody (Fig. 9B). Having established that P1 and P2 polyclonal antibody can bind in crude lysates to the GPA33 receptor, we next evaluated their response to DTT reduction using the purified GPA33 receptor domain (Fig. 9C). Although P1 polyclonal antibody bound to GPA33 in its oxidized or reduced state, the epitope for P2 polyclonal antibody was cryptic in the GPA33 protein, but its epitope was substantially exposed using DTT (Fig. 9C), thus exhibiting a mirror response to RSE-05 (Fig. 5C). The lower binding of P2 polyclonal antibody to oxidized GPA33 by ELISA (Fig. 9C) might explain the lower binding of P2 polyclonal antibody to the GPA33 receptor in the dot blot (Fig. 9B). The Atlas polyclonal antibody is stimulated by DTT treatment of GPA33 but still binds well to oxidized GPA33 (Fig. 5C).

We next compared the adaptability of RSE-05 in either capture and sensor formats, with the three antibodies: the commercially available Atlas polyclonal antibody, P1 polyclonal antibody (CPF1.5), and P2 polyclonal antibody (CEG2.6) (Fig. 9D). RSE-05 in the capture (solid) phase can only be used to detect GPA33 using P1 polyclonal antibody (CPF1.5) in the detection phase (Fig. 9D). Neither the Atlas polyclonal antibody, nor the P2 polyclonal antibody (CEG2.6), can bind well to RSE-05 captured GPA33 (Fig. 9D). By contrast, both the Atlas polyclonal antibody and the P1 polyclonal antibody (CPF1.5) can be used as capture agents and RSE-05 can detect GPA33 in the liquid phase (Fig. 9D). Altogether, these data indicate that RSE-05 can function in a lateral flow format and peptide 1 polyclonal antibody forms the superior pairing.

As additional controls, we also evaluated the ability of all three antibodies (RSE-05, P1 polyclonal antibody (CPF1.5) and P2 polyclonal antibody (CEG2.6)) to function in immunohistochemistry. Representative images in Supplementary Fig. 7 show, as in the ELISA, the RSE-05 and P2 polyclonal antibody (CEG2.6) have mutually exclusive binding characteristics. The P2 polyclonal antibody (CEG2.6)) only binds well to GPA33 protein using ELISA when the GPA33 protein is treated with DTT (Fig. 9C), but it also stains membrane material after antigen retrieval of formalin-fixed paraffin-embedded cancer tissue (Supplementary Fig. 7A). By contrast, RSE-05 does not bind to GPA33 using ELISA when the protein is treated with DTT (Fig. 5C), and it also does not stain cancer membrane material after antigen retrieval of formalin-fixed paraffin-embedded tissue (Supplementary Fig. 7B). These data might suggest that antigen retrieval can ‘open’ the P2 polyclonal antibody (CEG2.6) epitope but block the RSE-05 epitope. The P1 polyclonal antibody (CPF1.5) can bind to membrane material after antigen retrieval of formalin-fixed paraffin-embedded cancer tissue (Supplementary Fig. 7C). Altogether, these ELISA data highlight the utility of RSE-05 to detect oxidized GPA33 in combination with the P1 polyclonal antibody (CPF1.5) and that both antibodies can function in either solid or liquid phase state capture-sensor format.

## Discussion

### Target discovery in OAC for improving theranostic developments

Oesophageal adenocarcinoma is considered a cancer of high unmet clinical need owing to the paucity of effective therapeutic options. As such, developing novel therapeutic medicines is of high priority. One of the first modern antibody medicines trialed in patients with OAC, panitumumab (targeting EGFR), was evaluated because of the reported ~ 25–50% expression of EGFR in groups of patients with OAC [45]. However, the antibody in combination with standard chemotherapy decreased patient survival in a randomized, open-label phase 3 trial and was not recommended for use [13]. Although the panitumumab antibody was approved for use in colorectal cancer on the basis of clinical trial data [46, 47], it is unclear whether the opposing effects on patient survival in OAC versus colorectal cancer are due to immunological effects or whether positive feedback signalling on the EGFR pathway in cancer cells itself triggers cell growth [48]. In our dataset (Fig. 1A), EGFR did not exhibit enhanced tumour-specific expression, thus it is unclear at the bulk level of protein detected in mass spectrometric screens whether EGFR gene amplification correlates with enhanced EGFR protein production. ERBB2 antibody therapeutics also have an impact in OAC [12]. However, upon stratification using mass spectrometry, ERBB2 does not show noticeable tumour-specific expression (Fig. 1A). PD-L1-targeting immunotherapies have also formed antibody-based successes in oesophageal adenocarcinoma [14]. PD-L1 peptides were not detected in our tryptic mass spectrometric datasets [20]. However, it should be noted that PD-L1 antibody medicines often give positive results independent of PD-L1 status, possibly related to immune infiltration or the tumour mutational burden [49].

The Claudin 18.2 therapeutic zolbetuximab, in combination with capecitabine and oxaliplatin, can form compelling medicines for gastric or gastro-oesophageal junction cancers [50]. A related trial also demonstrated that zolbetuximab along with fluorouracil, folinic acid, and oxaliplatin can impact on a subclass of Claudin 18.2-positive/HER2-negative cancers [51]. Another significant advance in the treatment of upper GI cancers was the recent implementation of anti-Claudin 18.2 antibodies as an antibody drug conjugate (ADC) [52, 53]. As Claudin 18.2 expression can range from 17% to 29% in primary gastro-oesophageal junction tumours [54], with 26% of lymph nodes being Claudin 18.2 positive [55], additional targets are required. To identify novel membrane-based theranostic targets, we had carried out the largest multi-omics study in OAC [20]. Claudin 18.2 was not identified in this study; however, we identified GPA33 as a highly expressed cancer-tissue specific target (Fig. 1A) that also formed a novel class of targets by virtue of its high protein but low mRNA abundance [20]. Because Claudin 18.2 forms a new therapeutic benchmark [52, 53], it was necessary to determine whether GPA33 and Claudin 18.2 are co-expressed, independently expressed or negatively correlated. Such data would be important for therapeutic strategies, because if GPA33 was expressed in the same cells as Claudin 18.2, it would be difficult to rationalize developing a new GPA33 antibody therapeutic to compete with Claudin 18.2 antibodies.

Using a TMA from *n* = 106 cancer patients, we show here, unexpectedly, that GPA33 and Claudin 18.2 exhibit statistically significant mutually exclusive expression in primary cancer tissue cores (Fig. 1). The breakdown is as follows: 36% of cancers are GPA33^+^/Claudin 18.2^−^ whilst 22% are GPA33^−^/Claudin 18.2^+^ (Fig. 1H). The number of metastatic lymph nodes form a smaller component of the TMA, with *n* = 39 (Supplementary Fig. 2). Using this array, GPA33 and Claudin 18.2 also trend towards mutually exclusive expression in the lymph nodes; however, these numbers are not statistically significant (Supplementary Fig. 2E). Altogether, the data point towards the utility of stratifying patients for GPA33 and Claudin 18.2 for distinct therapeutics. In addition, as 33% of cancer cores are both GPA33 and Claudin 18.2 negative using immunohistochemistry (Fig. 1H), and most of our lymph node cores are also both GPA33 and Claudin 18.2 negative (Supplementary Fig. 2E), these data suggest that a ‘third’ set of therapeutics would be required to target this group of patients.

Because PD-L1 antibodies are also used clinically [14–16], we examined PD-L1 expression in our TMA to see how or whether PD-L1 expression related to GPA33^+^, Claudin 18.2^+^ or the dual-negative third group (Supplementary Fig. 1). However, we did not observe a high penetrance of PD-L1 protein expression in FFPE core material using the PD-L1 antibody E1L3N [56] (data not shown). Thus, we could not confidently determine whether GPA33 or Claudin 18.2 status is independent from or segregating with PD-L1. Therefore we do not think PD-L1 necessarily captures this ‘third’ group of GPA33^-^/Claudin18.2^-^ patients. It should also be emphasized that a tissue ‘core’ used to form a TMA can be composed of a relatively small (~ 5–10 μm × ~ 1 mm) slice, and does not capture the full landscape of the much larger resected, FFPE embedded cancer tissue block which can be several millimetres in diameter. By using larger tissue blocks, we might be able to better define PD-L1 status. In addition, by increasing the surface area of tissue analysed, it is possible that both GPA33 and Claudin 18.2 double-negative cancers drop out and that one or the other might be expressed. It would be interesting in the future to carry out multiomics dissection of GPA33/Claudin 18.2 dual-negative cancers to identify components of pathways that drive the formation of this distinct group. Such an analysis might identify additional membrane targets, small molecule targets or classic kinase targets whose inhibition might augment immunotherapies.

In addition to the possibility that GPA33 and Claudin 18.2 represent two distinct OAC developmental paths forming two therapeutic groups (Supplementary Fig. 1), GPA33 exhibits superior tumour-specific expression compared with Claudin 18.2, the latter of which is expressed at membranes in normal gastric tissue in most of our patient samples (Fig. 1G). The high expression of Claudin 18.2 in normal gastric tissue has been noted [36] and speculated to be related to gastric toxicity of anti-Claudin 18.2 therapeutics. Thus, on the diagnostic front, GPA33, but not Claudin 18.2, could form the basis for a cancer diagnostic that captures ~  > 1/3 of cancer patients (Supplementary Fig. 1). These immunohistochemical data are consistent with the mass spectrometric data demonstrating that Claudin 18.2 peptides do not exhibit ‘tumour-specific enrichment’ (Fig. 1A).

A strategic question can be raised, therefore, as to whether targets with selective expression in cancer tissue might be the best inclusion criteria. As stated above, anti-PD-L1 antibodies also appear to function independent of PD-L1 status in some situations [49]. Similarly, although Claudin 18.2 monoclonal antibodies represent the most recent targeted therapy being trialled in upper GI cancers, with positive effects seen in patients [50, 52], Claudin 18.2 does not show substantial over-production in tumour tissue versus normal gastric tissue using immunohistochemistry (Fig. 1G). The reason Claudin 18.2 is proposed to be amenable as a target, despite the high expression in normal gastric tissue, is that its epitope is available in the relatively disordered tumour ultrastructure, whilst in normal gastric tissue, Claudin 18.2 is within a tight junction and possibly concealed. So absolute target abundance, or cancer-specific expression, does not necessarily translate to target availability, although there remains gastric toxicity from some Claudin 18.2 antibody treatments [36, 57].

### Antibody-based diagnostics in OAC

Therapeutic tools remain one aim of our work; however, the cancer specificity of GPA33, as opposed to Claudin 18.2, could support the development of cancer-specific diagnostics. GPA33 is one of almost ten receptor targets that we considered for potential diagnostic and/or imaging in OAC on the basis of abundance in OAC tissue compared with two normal adjacent tissues, squamous oesophagus and gastric epithelium [20] (Fig. 1A). We first focussed on GPA33 given its relatively high patient penetrance (Fig. 1), and we developed a lead monoclonal antibody to this target using scFV phage display. In this report, we focus not on whether such a monoclonal antibody could, for example, be used as a therapeutic tool in an ADC format to kill GPA33^+^ cancer cells. Rather, we focus on whether we can develop a diagnostic prototype assay in an ELISA format that can measure GPA33 in solution as a lateral flow surrogate. Such knowledge could be used to invent a lateral flow device to rapidly measure GPA33^+^ cancers in endoscopic biopsies or to produce fluorescently labelled MABs for image-guided resection of oesophageal lesions to better identify cancer margins and/or otherwise not visible micro-metastases. Prior to our work, one of the most dominating antibodies to GPA33 was derived from a murine hybridoma and humanized for use in imaging colorectal cancer [58, 59]. In addition, recently a GOLD scFV library was used to acquire antibodies to GPA33 as therapeutics tools that can induce cell death in colorectal cancer cell models [60].

Many antibody sequences were identified after biopanning using NGS sequencing data (Fig. 2 and Supplementary Table 1). We did not clone out the rarer antibody sequences, but rather focussed on the single most abundant sequence (Fig. 2E). An interesting feature of the RSE-05 antibody is that its epitope appears to be constrained by di-sulphide bonds (Figs. 5 and 6). Many extracellular receptors are composed of multiple di-sulphide bridges which form a type of combination lock that forms the bioactive structure of the molecule. An example is the epithelial cell adhesion molecule (EPCAM) receptor, whose structure was solved after production in eukaryotic insect cell expression systems [61], which allows its entry into the endoplasmic reticulum where, under pro-oxidizing conditions, the protein can be folded by chaperones such as the oncogenic protein di-sulphide isomerase (PDI), AGR2 [62]. Indeed, GPA33 apparently contains three di-sulphide pairs (Fig. 6), which implies the requirement of a protein di-sulphide isomerase chaperone system to facilitate folding and processing to the plasma membrane. Although the non-denaturing dot blot (Fig. 6) identified the most sensitive di-sulphide formed by Cys22 and Cys96 in ‘lobe 1’ as a dominant feature of the epitope, reducing and denaturing immunoblotting revealed that mutation of any one of the other four cysteine residues in ‘lobe 2’ also inhibited antibody binding using SDS-PAGE (Fig. 6). These data suggest that allosteric interactions from ‘SDS denaturation’ of lobe 2, due to a single Cys–Ser lobe 2 mutation, can impact on the primary epitope in lobe 1, itself constrained by Cys22 and Cys96 di-sulphide. Interestingly, XL-MS (Fig. 8) identified one intra- or interdomain covalent bond between lobe 1 and lobe 2 between Ser2 and Lys122 (Fig. 8B, XL-4), suggesting that, in the GPA33 protein preparation we used, a proportion of the protein exists in a different solution conformation than that defined by Colabfold [44] in Fig. 6A; the ‘extended’ structure in Fig. 6A predicts that lobe 1 and lobe 2 are contiguous, and with no proximity evident between lobe 1 and lobe 2. The combination of cross-linking mass spectrometry (Fig. 8) along with a study of the effects of Cys–Ser mutations on RSE-05 binding (Fig. 6) suggests that allosteric interactions between lobe 1 and lobe 2 might impact on the primary epitope of RSE-05 in lobe 1.

Because the features of a monoclonal antibody epitope, such as RSE-05, whose binding to its cognate antigen is inhibited directly by di-sulphide reduction, is rarely reported, we focussed in this manuscript on characterizing the epitope of RSE-05. By contrast to RSE-05, most commercially available antibodies used for research purposes have associated documentations indicating that they can be used in a denaturing immunoblot with reducing agents such as DTT or β-mercaptoethanol. For example, the commercially available ‘Atlas’ antibody used for the GPA33 ELISA is stimulated by DTT (Fig. 5C), which is consistent with its compatibility with denaturation and reducing immunoblots (Fig. 6C). The few, prior examples of antibodies whose epitopes are sensitive to di-sulphide-bridge disruption include a malarial protein [63], the insulin receptor [64] and CD38 [43]. In addition, using the same scFV-phage library used in this manuscript [28], we have generated an antibody that binds to a linear epitope in the PD-1 receptor [28] but also a novel scFV-derived monoclonal antibody generated towards T-cell immunoglobulin and mucin domain-containing molecule 3 (TIM-3) that has an epitope which is disrupted by prior reduction (manuscript submitted). Thus, using this scFV phage display library, we now have isolated two receptors out of three (TIM-3 and GPA33) whose epitopes are sensitive to reduction.

We find the property of monoclonal antibodies with di-sulphide stabilized, conformational epitopes interesting because it raises a question about the diversity of antibody genes that exist in the naïve B-cell repertoire of a mammal. Because generation of antibodies used for most research purposes immunizes an antigen with adjuvant into animals [65], possibly denaturing the antigen, we can envision that this often results in the acquisition of antibodies that bind linear, non-conformational epitopes. Because the antibody library we generated does not come from immunized animals [28], but from a cloned naïve B-cell repertoire, this means that antibody folds that can contact di-sulphide-constrained conformational epitopes in proteins such as GPA33 or TIM-3 are pre-existing in vivo. It might be that such antibodies are more common than is realized because the general bias in antibody production is to immunize animals with possibly denaturing adjuvants, meaning that the field tends to acquire antibodies that bind to linear non-conformational epitopes. For example, a recent study examining the high-resolution structure of antigens bound to antibodies focussed on ~ 200 structures of antibodies bound to peptides [66]. If conformationally sensitive antibodies are more commonly existing in nature than is realized, then it is possible that the phage biopanning screening method can have a positive impact on identifying such antibodies because intact structural proteins can be used for screening. Indeed, we have found, that if we switch our biopanning selection method, from using recombinant GPA33 protein to using linear peptides derived from GPA33, we can isolate scFV antibodies that bind to non-conformational linear epitopes (manuscript in preparation). These data also indicate that pre-existing anti-GPA33 antibodies are present in our scFv library that can contact linear epitopes. Such antibodies might have been enriched during biopanning, such as those summarized in Supplementary Table 1 and Fig. 2E.

Although endoscopic imaging using fluorescently labelled GPA33 antibody is one potential outcome of its clinical utility, a lateral flow device for quantitation from liquid biopsies typically requires a ‘capture’ antibody and a ‘sensor’ or detection antibody. As the RSE-05 antibody captures an epitope in ‘lobe 1’ of the protein (Fig. 8), we sought to ask whether RSE-05 is in fact adaptable in a capture-sensor format. For example, RSE-05 might sterically hinder other antibodies that function in a capture-sensor format, and/or it is possible that other antibodies would have epitopes requiring full denaturation (i.e. reduction) of GPA33 that would in turn be incompatible with RSE-05. This would rule out RSE-05 as a diagnostic in lateral flow format. Three polyclonal antibodies were tested in capture-sensor formats with RSE-05 (Fig. 9). Only one polyclonal antibody (P1 CPF1.5) out of the three tested could be used in combination with RSE-05 in both capture-sensor orientations (Fig. 9D). The Atlas polyclonal antibody, by contrast, could not be used with RSE-05 in the capture mode (Fig. 9D). This might be because the Atlas antibody epitope is stimulated by DTT and RSE-05 might lock GPA33 in the oxidized isoform, thus precluding Atlas antibody binding in the detection mode. By contrast, the Atlas antibody can capture GPA33, and this can still be detected by RSE-05 in the detection mode. These data suggest that additional monoclonal antibody development towards GPA33 will likely yield an optimized monoclonal antibody pairing useful for lateral flow measurement.

## Conclusions

Our data provide an approach for shortlisting candidate cancer-specific protein receptors in OAC using mass spectrometric data [20] and pathology analysis that would form the basis for future diagnostics and therapeutics. Since OAC can be thought of as a gastro-oesophageal junction tumour, the validation of any such target requires analysis of expression of any given target in normal adjacent tissues, both squamous oesophagus and glandular gastric (Fig. 1). The GPA33 receptor itself is tumour specific as defined using either mass spectrometry (Fig. 1A) or immunohistochemistry in two normal adjacent tissues from the same patient (Fig. 1G). We also present data (Fig. 1 and Supplementary Fig. 2) suggesting that GPA33 can capture a diagnostic subclass of OAC distinct from Claudin 18.2. In addition, the GPA33 protein fulfils another key criterion including its prior detection as a biomarker of Barrett’s oesophagus [21] and GPA33 exhibits superior cancer specificity compared with Claudin 18.2 as a diagnostic. We also highlight the utility of using non-denatured and eukaryotically expressed GPA33 receptor domain as a bait in a scFv phage encoded antibody library screen, where we also include next-generation CDR3 sequencing to follow the efficiency of biopanning and allowing the identification of lower-abundance antibodies not identified using classic biopanning by ELISA (Fig. 2). The lead antibody generated, which binds to a redox-sensitive conformational epitope in GPA33, provides a biologic tool for validation as a diagnostic imaging tool in living cancer cells (Fig. 4), in lateral flow format using biopsies from endoscopy for rapid evaluation (Fig. 9) and possibly as a therapeutic in oesophageal adenocarcinoma. Since OAC is apparently composed of at least three different cancer sub-classes, with respect to GPA33 and Claudin 18.2 receptor status (Supplementary Fig. 1), additional targets would be required to fully capture the cancer barcode of OAC in a diagnostic and therapeutic setting.

## Supplementary Information

Supplementary material 1.

Supplementary material 2.

## Data Availability

Mass spectra were uploaded to PRIDE; Dataset identifier: PXD060098; Username: reviewer_pxd060098@ebi.ac.uk; Password: wTsh64h4DTRe. The gels and immunoblots are digital and represent the original non cropped images taken using digital image capture, except for Fig. 3E and Fig. 4A which are shown in supplemental material.

## Abbreviations

OAC: Oesophageal adenocarcinoma
NGS: Next-generation DNA sequencing
OSCC: Oesophageal squamous cell carcinoma
HRP: Horseradish peroxidase
PBS: Phosphate-buffered saline
DTT: Dithiothreitol
SDS: Sodium dodecyl sulfate
PEI: Polyethylene immine
PBST: Phosphate-buffered saline plus Tween 20
BSA: Bovine serum albumin
ACN: Acetonitrile
MS: Mass spectrometry
TOF: Time of flight
IHC: Immunohistochemistry
TMA: Tumour microarray
GI: Gastrointestinal
ScFV: Single-chain antibody fragment variable domain
FR4: Framework 4 antibody domain
CDR3: The third complementarity-determining region antibody domain
Fc: Fragment crystallizable region antibody domain
XL-MS: Cross-linking mass spectrometry
IgG: Immunoglobulin antibody
DSS: Disuccinimidyl suberate
EGS: Ethylene glycol bis(succinimidyl succinate)

## References

[1] Thrift AP. Global burden and epidemiology of Barrett oesophagus and oesophageal cancer. Nat Rev Gastroenterol Hepatol. 2021;18(6):432–43. 33603224 10.1038/s41575-021-00419-3

[2] Zhang X, Peng L, Luo Y, Zhang S, Pu Y, Chen Y, et al. Dissecting esophageal squamous-cell carcinoma ecosystem by single-cell transcriptomic analysis. Nat Commun. 2021;12(1):5291. 34489433 10.1038/s41467-021-25539-x PMC8421382

[3] Savarino E, de Bortoli N, De Cassan C, Della Coletta M, Bartolo O, Furnari M, et al. The natural history of gastro-esophageal reflux disease: a comprehensive review. Dis Esophagus. 2017;30(2):1–9. 27862680 10.1111/dote.12511

[4] Killcoyne S, Fitzgerald RC. Evolution and progression of Barrett’s oesophagus to oesophageal cancer. Nat Rev Cancer. 2021;21(11):731–41. 34545238 10.1038/s41568-021-00400-x

[5] Honing J, Tan WK, Lu VYZ, Gourgiotis V, Gianfrancesco IM, Schumacher AA, et al. Surveillance of Barrett’s esophagus patients in an expert center is associated with low disease‐specific mortality. United Eur Gastroenterol J. 2025;13(2):220–8. 10.1002/ueg2.12759 PMC11975633 39949075

[6] Gourgiotis V, Graham C, Foerster K, Fitzgerald RC, Harvey R, NHS England Pilot and NHS England Oversight Group, et al. Use of a non-endoscopic capsule-sponge triage test for reflux symptoms: results from the NHS England prospective real-world evaluation. Aliment Pharmacol Ther. 2025;61(5):876–85. 39794909 10.1111/apt.18472 PMC11825927

[7] Weaver JMJ, Ross-Innes CS, Shannon N, Lynch AG, Forshew T, Barbera M, et al. Ordering of mutations in preinvasive disease stages of esophageal carcinogenesis. Nat Genet. 2014;46(8):837–43. 24952744 10.1038/ng.3013 PMC4116294

[8] Ross-Innes CS, Chettouh H, Achilleos A, Galeano-Dalmau N, Debiram-Beecham I, MacRae S, et al. Risk stratification of Barrett’s oesophagus using a non-endoscopic sampling method coupled with a biomarker panel: a cohort study. Lancet Gastroenterol Hepatol. 2017;2(1):23–31. 28404010 10.1016/S2468-1253(16)30118-2

[9] Bolger JC, Donohoe CL, Lowery M, Reynolds JV. Advances in the curative management of oesophageal cancer. Br J Cancer. 2022;126(5):706–17. 34675397 10.1038/s41416-021-01485-9 PMC8528946

[10] Rustgi AK, El-Serag HB. Esophageal Carcinoma. N Engl J Med. 2014;371(26):2499–509. 25539106 10.1056/NEJMra1314530

[11] Napier KJ, Scheerer M, Misra S. Esophageal cancer: a review of epidemiology, pathogenesis, staging workup and treatment modalities. World J Gastrointest Oncol. 2014;6(5):112–20. 24834141 10.4251/wjgo.v6.i5.112 PMC4021327

[12] Bang YJ, Van Cutsem E, Feyereislova A, Chung HC, Shen L, Sawaki A, et al. Trastuzumab in combination with chemotherapy versus chemotherapy alone for treatment of HER2-positive advanced gastric or gastro-oesophageal junction cancer (ToGA): a phase 3, open-label, randomised controlled trial. Lancet. 2010;376(9742):687–97. 20728210 10.1016/S0140-6736(10)61121-X

[13] Waddell T, Chau I, Cunningham D, Gonzalez D, Okines AFC, Okines C, et al. Epirubicin, oxaliplatin, and capecitabine with or without panitumumab for patients with previously untreated advanced oesophagogastric cancer (REAL3): a randomised, open-label phase 3 trial. Lancet Oncol. 2013;14(6):481–9. 23594787 10.1016/S1470-2045(13)70096-2 PMC3669518

[14] Zhao Y, Tsujimoto A, Ide T, Zhang J, Feng Y, Gao L, et al. Model-based population pharmacokinetic and exposure response analyses for safety and efficacy of nivolumab as adjuvant treatment in subjects with resected oesophageal or gastroesophageal junction cancer. Br J Clin Pharmacol. 2024;90(11):2920–30. 39054780 10.1111/bcp.16188

[15] Wang C, Xie Q, Miao Y, He W, Wang K, Liu G, et al. Real-world efficacy of PD-1 inhibitors in treating oesophageal squamous cell carcinoma (ESCAPE): protocol of a multicentre, real-world, observational, cohort study. BMJ Open. 2025;15(10):e098524. 41125270 10.1136/bmjopen-2024-098524 PMC12548579

[16] Lin Y, Liang HW, Liu Y, Pan XB. Nivolumab adjuvant therapy for esophageal cancer: a review based on subgroup analysis of CheckMate 577 trial. Front Immunol. 2023;14:1264912. 37860010 10.3389/fimmu.2023.1264912 PMC10582756

[17] Angerilli V, Ghelardi F, Nappo F, Grillo F, Parente P, Lonardi S, et al. Claudin-18.2 testing and its impact in the therapeutic management of patients with gastric and gastroesophageal adenocarcinomas: a literature review with expert opinion. Pathol Res Pract. 2024;254:155145. 38277741 10.1016/j.prp.2024.155145

[18] Hong JY, An JY, Lee J, Park SH, Park JO, Park YS, et al. Claudin 18.2 expression in various tumor types and its role as a potential target in advanced gastric cancer. Transl Cancer Res. 2020;9(5):3367–74. 35117702 10.21037/tcr-19-1876 PMC8797704

[19] O’Neill JR, Pak HS, Pairo-Castineira E, Save V, Paterson-Brown S, Nenutil R, et al. Quantitative shotgun proteomics unveils candidate novel Esophageal Adenocarcinoma (EAC)-specific Proteins. Mol Cell Proteomics. 2017;16(6):1138–50. 28336725 10.1074/mcp.M116.065078 PMC5461543

[20] O’Neill JR, Yébenes Mayordomo M, Mitulović G, Al Shboul S, Bedran G, Faktor J, et al. Multi-omic analysis of esophageal adenocarcinoma uncovers candidate therapeutic targets and cancer-selective posttranscriptional regulation. Mol Cell Proteomics. 2024;23(6):100764. 38604503 10.1016/j.mcpro.2024.100764 PMC11245951

[21] Wong N, Warren BF, Piris J, Maynard N, Marshall R, Bodmer WF. EpCAM and gpA33 are markers of Barrett’s metaplasia. J Clin Pathol. 2006;59(3):260–3. 16473928 10.1136/jcp.2005.027474 PMC1860330

[22] Wilson CH, McIntyre RE, Arends MJ, Adams DJ. The activating mutation R201C in GNAS promotes intestinal tumourigenesis in Apc(Min/+) mice through activation of Wnt and ERK1/2 MAPK pathways. Oncogene. 2010;29(32):4567–75. 20531296 10.1038/onc.2010.202 PMC2923080

[23] Rageul J, Mottier S, Jarry A, Shah Y, Théoleyre S, Masson D, et al. KLF4-dependent, PPARgamma-induced expression of GPA33 in colon cancer cell lines. Int J Cancer. 2009;125(12):2802–9. 19551868 10.1002/ijc.24683 PMC2791338

[24] Morgana F, Opstelten R, Slot MC, Scott AM, van Lier RAW, Blom B, et al. Single-cell transcriptomics reveals discrete steps in regulatory T cell development in the human thymus. J Immunol. 2022;208(2):384–95. 34937744 10.4049/jimmunol.2100506

[25] Solmi R, De Sanctis P, Zucchini C, Ugolini G, Rosati G, Del Governatore M, et al. Search for epithelial-specific mRNAs in peripheral blood of patients with colon cancer by RT-PCR. Int J Oncol. 2004;25(4):1049–56. 15375555

[26] Cheal SM, McDevitt MR, Santich BH, Patel M, Yang G, Fung EK, et al. Alpha radioimmunotherapy using ^225^Ac-proteus-DOTA for solid tumors - safety at curative doses. Theranostics. 2020;10(25):11359–75. 33052220 10.7150/thno.48810 PMC7546012

[27] Wu Z, Guo HF, Xu H, Cheung NKV. Development of a tetravalent anti-GPA33/anti-CD3 bispecific antibody for colorectal cancers. Mol Cancer Ther. 2018;17(10):2164–75. 30082472 10.1158/1535-7163.MCT-18-0026 PMC6168351

[28] Lisowska M, Worrall EG, Zavadil-Kokas F, Charlton K, Murray E, Mohtar MA, et al. The development of a canine single-chain phage antibody library to isolate recombinant antibodies for use in translational cancer research. Cell Rep Methods. 2025;5(3):101008. 40132540 10.1016/j.crmeth.2025.101008 PMC12049728

[29] Hrabal V, Stenckova M, Zavadil Kokas F, Muller P, Nenutil R, Vojtesek B, et al. TAp73 and ΔTAp73 isoforms show cell-type specific distributions and alterations in cancer. Sci Rep. 2024;14:29949. 39622910 10.1038/s41598-024-80927-9 PMC11612387

[30] Krejci A, Hupp TR, Lexa M, Vojtesek B, Muller P. Hammock: a hidden Markov model-based peptide clustering algorithm to identify protein-interaction consensus motifs in large datasets. Bioinformatics. 2016;32(1):9–16. 26342231 10.1093/bioinformatics/btv522 PMC4681989

[31] Hammond JB, Kruger NJ. The bradford method for protein quantitation. Methods Mol Biol. 1988;3:25–32. 21400151 10.1385/0-89603-126-8:25

[32] Singh A, Padariya M, Faktor J, Kote S, Mikac S, Dziadosz A, et al. Identification of novel interferon responsive protein partners of human leukocyte antigen A (HLA-A) using cross-linking mass spectrometry (CLMS) approach. Sci Rep. 2022;12(1):19422. 36371414 10.1038/s41598-022-21393-z PMC9653400

[33] Nowicki-Osuch K, Zhuang L, Cheung TS, Black EL, Masqué-Soler N, Devonshire G, et al. Single-cell RNA sequencing unifies developmental programs of esophageal and gastric intestinal metaplasia. Cancer Discov. 2023;13(6):1346–63. 36929873 10.1158/2159-8290.CD-22-0824 PMC10236154

[34] Moore PA, Shah K, Yang Y, Alderson R, Roberts P, Long V, et al. Development of MGD007, a gpA33 x CD3-bispecific DART protein for T-cell immunotherapy of metastatic colorectal cancer. Mol Cancer Ther. 2018;17(8):1761–72. 29866746 10.1158/1535-7163.MCT-17-1086

[35] Vaughn BA, Lee SG, Vargas DB, Seo S, Rinne SS, Xu H, et al. Theranostic GPA33-pretargeted radioimmunotherapy of human colorectal carcinoma with a bivalent ^177^Lu-labeled radiohapten. J Nucl Med. 2024;65(10):1611–8. 39168519 10.2967/jnumed.124.267685 PMC11448610

[36] Carstens EJ, Takahashi K, Sakamoto N, De Vizio M, Morgado M, Nikkhoi SK, et al. Modeling and addressing on-target/off-tumor toxicity of claudin 18.2 targeted immunotherapies. Nat Commun. 2025;16(1):9651. 41176582 10.1038/s41467-025-65148-6 PMC12579598

[37] Qi C, Liu C, Peng Z, Zhang Y, Wei J, Qiu W, et al. Claudin-18 isoform 2-specific CAR T-cell therapy (satri-cel) versus treatment of physician’s choice for previously treated advanced gastric or gastro-oesophageal junction cancer (CT041-ST-01): a randomised, open-label, phase 2 trial. Lancet. 2025;405(10494):2049–60. 40460847 10.1016/S0140-6736(25)00860-8

[38] Rohde C, Yamaguchi R, Mukhina S, Sahin U, Itoh K, Türeci Ö. Comparison of Claudin 18.2 expression in primary tumors and lymph node metastases in Japanese patients with gastric adenocarcinoma. Jpn J Clin Oncol. 2019;49(9):870–6. 31087075 10.1093/jjco/hyz068 PMC6792344

[39] Cheal SM, Xu H, Guo HF, Lee SG, Punzalan B, Chalasani S, et al. Theranostic pretargeted radioimmunotherapy of colorectal cancer xenografts in mice using picomolar affinity ^86^Y- or ^177^Lu-DOTA-Bn binding scFv C825/GPA33 IgG bispecific immunoconjugates. Eur J Nucl Med Mol Imaging. 2016;43(5):925–37. 26596724 10.1007/s00259-015-3254-8 PMC4814317

[40] Yoshimoto S, Chester N, Xiong A, Radaelli E, Wang H, Brillantes M, et al. Development and pharmacokinetic assessment of a fully canine anti-PD-1 monoclonal antibody for comparative translational research in dogs with spontaneous tumors. MAbs. 2023;15(1):2287250. 38047502 10.1080/19420862.2023.2287250 PMC10793675

[41] Bobrovnik SA. Determination of antibody affinity by ELISA. Theory. J Biochem Biophys Methods. 2003;57(3):213–36. 14512156 10.1016/s0165-022x(03)00145-3

[42] Heinrich L, Tissot N, Hartmann DJ, Cohen R. Comparison of the results obtained by ELISA and surface plasmon resonance for the determination of antibody affinity. J Immunol Methods. 2010;352(1–2):13–22. 19854197 10.1016/j.jim.2009.10.002

[43] Wagner FF. Antibody testing in patients treated with anti-CD38: there is still room for improvement. Blood Transfus. 2020;18(4):244–6. 32697927 10.2450/2020.0166-20 PMC7375888

[44] Mirdita M, Schütze K, Moriwaki Y, Heo L, Ovchinnikov S, Steinegger M. ColabFold: making protein folding accessible to all. Nat Methods. 2022;19(6):679–82. 35637307 10.1038/s41592-022-01488-1 PMC9184281

[45] Langer R, Von Rahden BHA, Nahrig J, Von Weyhern C, Reiter R, Feith M, et al. Prognostic significance of expression patterns of c-erbB-2, p53, p16INK4A, p27KIP1, cyclin D1 and epidermal growth factor receptor in oesophageal adenocarcinoma: a tissue microarray study. J Clin Pathol. 2006;59(6):631–4. 16731604 10.1136/jcp.2005.034298 PMC1860401

[46] Chu E. Panitumumab: a new anti-EGFR antibody for the treatment of advanced colorectal cancer. Clin Colorectal Cancer. 2006;6(1):13. 16796785 10.3816/CCC.2006.n.016

[47] Gibson TB, Ranganathan A, Grothey A. Randomized phase III trial results of panitumumab, a fully human anti-epidermal growth factor receptor monoclonal antibody, in metastatic colorectal cancer. Clin Colorectal Cancer. 2006;6(1):29–31. 16796788 10.3816/CCC.2006.n.01

[48] Georgopoulos NT, Kirkwood LA, Southgate J. A novel bidirectional positive-feedback loop between Wnt-β-catenin and EGFR-ERK plays a role in context-specific modulation of epithelial tissue regeneration. J Cell Sci. 2014;127(Pt 13):2967–82. 24816560 10.1242/jcs.150888 PMC4077591

[49] Ilson DH. Immunotherapy in esophagogastric cancer. Clin Adv Hematol Oncol. 2021;19(10):639–47. 34637430 PMC9585692

[50] Shah MA, Shitara K, Ajani JA, Bang YJ, Enzinger P, Ilson D, et al. Zolbetuximab plus CAPOX in CLDN18.2-positive gastric or gastroesophageal junction adenocarcinoma: the randomized, phase 3 GLOW trial. Nat Med. 2023;29(8):2133–41. 37524953 10.1038/s41591-023-02465-7 PMC10427418

[51] Shitara K, Lordick F, Bang YJ, Enzinger P, Ilson D, Shah MA, et al. Zolbetuximab plus mFOLFOX6 in patients with CLDN18.2-positive, HER2-negative, untreated, locally advanced unresectable or metastatic gastric or gastro-oesophageal junction adenocarcinoma (SPOTLIGHT): a multicentre, randomised, double-blind, phase 3 trial. Lancet. 2023;401(10389):1655–68. 37068504 10.1016/S0140-6736(23)00620-7

[52] Liu J, Yang J, Sun Y, Gong J, Yue J, Pan Y, et al. CLDN18.2–targeting antibody–drug conjugate IBI343 in advanced gastric or gastroesophageal junction adenocarcinoma: a phase 1 trial. Nat Med. 2025;31(9):3028–36. 40670773 10.1038/s41591-025-03783-8 PMC12443601

[53] Klempner SJ, Sundar R. Evolution of claudin18.2 therapies in gastroesophageal cancers. Nat Med. 2025;31(9):2861–2. 40830662 10.1038/s41591-025-03898-y

[54] Coati I, Lotz G, Fanelli GN, Brignola S, Lanza C, Cappellesso R, et al. Claudin-18 expression in oesophagogastric adenocarcinomas: a tissue microarray study of 523 molecularly profiled cases. Br J Cancer. 2019;121(3):257–63. 31235864 10.1038/s41416-019-0508-4 PMC6738069

[55] Arnold A, Daum S, von Winterfeld M, Berg E, Hummel M, Rau B, et al. Prognostic impact of Claudin 18.2 in gastric and esophageal adenocarcinomas. Clin Transl Oncol. 2020;22(12):2357–63. 32488802 10.1007/s12094-020-02380-0 PMC7577914

[56] Song L, Zeng L, Yan H, Xu Q, Xia Q, Lei J, et al. Validation of E1L3N antibody for PD-L1 detection and prediction of pembrolizumab response in non-small-cell lung cancer. Commun Med (Lond). 2022;2(1):137. 36352254 10.1038/s43856-022-00206-4 PMC9626637

[57] Tojjari A, Idrissi YA, Saeed A. Emerging targets in gastric and pancreatic cancer: focus on claudin 18.2. Cancer Lett. 2024;611:217362. 39637967 10.1016/j.canlet.2024.217362

[58] Welt S, Divgi CR, Real FX, Yeh SD, Garin-Chesa P, Finstad CL, et al. Quantitative analysis of antibody localization in human metastatic colon cancer: a phase I study of monoclonal antibody A33. J Clin Oncol. 1990;8(11):1894–906. 2230877 10.1200/JCO.1990.8.11.1894

[59] King DJ, Antoniw P, Owens RJ, Adair JR, Haines AM, Farnsworth AP, et al. Preparation and preclinical evaluation of humanised A33 immunoconjugates for radioimmunotherapy. Br J Cancer. 1995;72(6):1364–72. 8519646 10.1038/bjc.1995.516 PMC2034099

[60] Murer P, Plüss L, Neri D. A novel human monoclonal antibody specific to the A33 glycoprotein recognizes colorectal cancer and inhibits metastasis. MAbs. 2020;12(1):1714371. 31928310 10.1080/19420862.2020.1714371 PMC6999842

[61] Pavšič M, Gunčar G, Djinović-Carugo K, Lenarčič B. Crystal structure and its bearing towards an understanding of key biological functions of EpCAM. Nat Commun. 2014;5:4764. 25163760 10.1038/ncomms5764

[62] Mohtar MA, Hernychova L, O’Neill JR, Lawrence ML, Murray E, Vojtesek B, et al. The sequence-specific peptide-binding activity of the protein sulfide isomerase AGR2 directs its stable binding to the oncogenic receptor EpCAM. Mol Cell Proteomics. 2018;17(4):737–63. 29339412 10.1074/mcp.RA118.000573 PMC5880107

[63] Coley AM, Campanale NV, Casey JL, Hodder AN, Crewther PE, Anders RF, et al. Rapid and precise epitope mapping of monoclonal antibodies against *Plasmodium falciparum* AMA1 by combined phage display of fragments and random peptides. Protein Eng. 2001;14(9):691–8. 11707616 10.1093/protein/14.9.691

[64] Jacobs S, Cuatrecasas P. Disulfide reduction converts the insulin receptor of human placenta to a low affinity form. J Clin Invest. 1980;66(6):1424–7. 7440724 10.1172/JCI109996 PMC371629

[65] Laustsen AH, Greiff V, Karatt-Vellatt A, Muyldermans S, Jenkins TP. Animal immunization, *in vitro* display technologies, and machine learning for antibody discovery. Trends Biotechnol. 2021;39(12):1263–73. 33775449 10.1016/j.tibtech.2021.03.003

[66] Lee JH, Yin R, Ofek G, Pierce BG. Structural features of antibody-peptide recognition. Front Immunol. 2022;13:910367. 35874680 10.3389/fimmu.2022.910367 PMC9302003

[67] Kemmer G, Keller S. Nonlinear least-squares data fitting in Excel spreadsheets. Nat Protoc. 2010;5(2):267–81. 20134427 10.1038/nprot.2009.182

