# Supplementary material for: GPA33 forms a distinct diagnostic target class to Claudin 18.2 in oesophageal adenocarcinoma enabling the development of a novel GPA33 antibody-based detection platform
Source: Cell Mol Biol Lett. 2026 Mar 10;31:58. doi: 10.1186/s11658-025-00852-1 (PMC13088578; doi:10.1186/s11658-025-00852-1)
Supplement: Supplementary file 1 — Supplementary material 1. [file 11658_2025_852_MOESM1_ESM.docx]

*SUPPLEMENTARY FIGURES*

*SF1.*

*
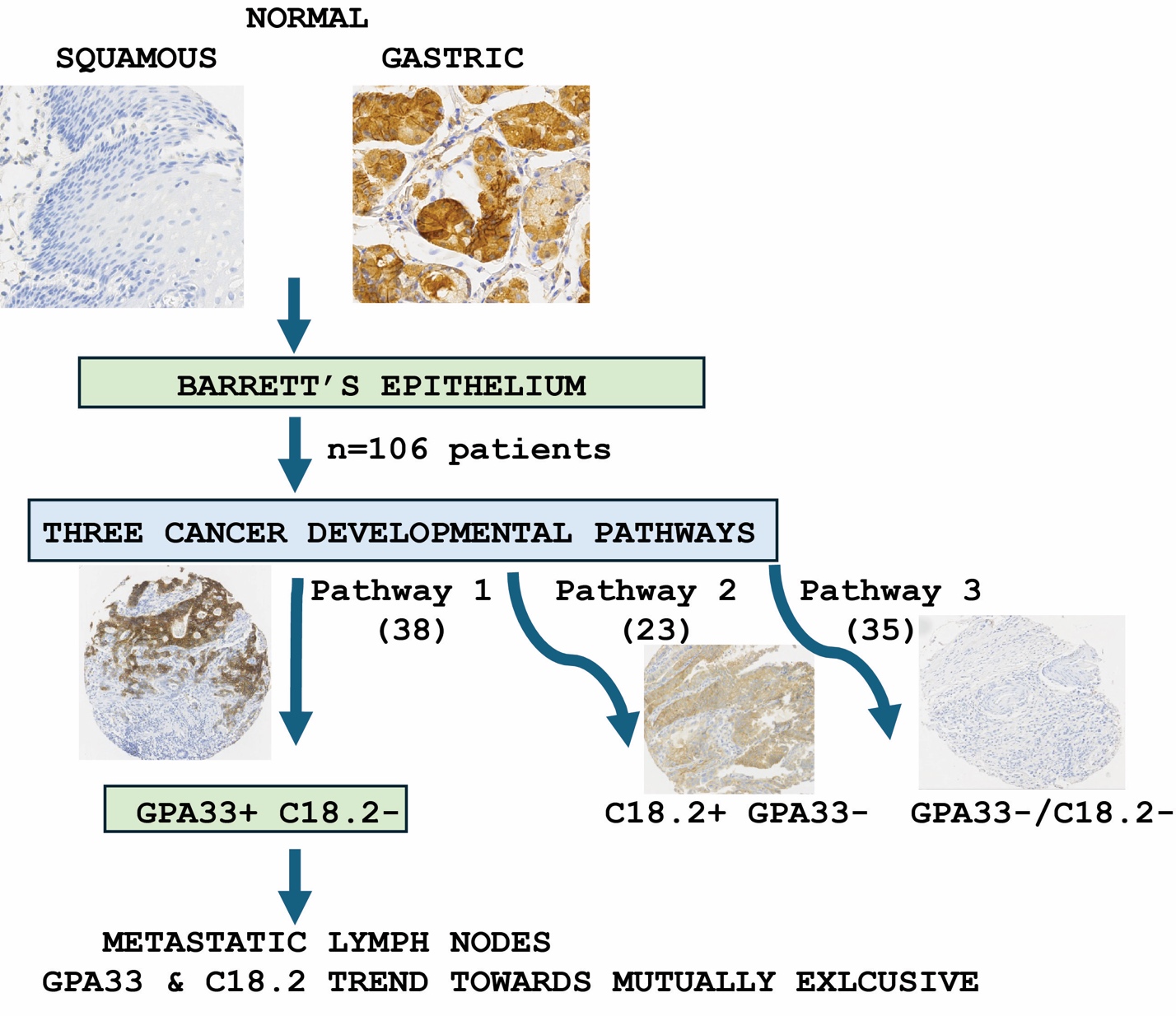
*

***SFigure 1. The flow chart summarizes the main clinically relevant expression data on GPA33 and Claudin18.2 in the tumour microarray cores.*** *Barrett’s Epithelium emerges from the junctional gastro-oesophageal region linked to acid reflux disease. Barrett’s epithelium is GPA33 +. Based on the generally mutually exclusive expression of GPA33 and Claudin18.2 in primary tumours (Figures 1H), we can propose that there are three distinct OAC cancer progression pathways or endpoint tumorigenic states-the first is GPA33+/ Claudin18.2-, the second is Claudin18.2+/GPA33-, and the third is negative for both GPA33 and Claudin18.2. Cores from metastatic lymph nodes obey a similar trend; mutually exclusive expression of GPA33 and Claudin18.2 (SFigure 2). However, there are some cancer cores which appear to express both GPA33 and Claudin18.2 in the same cell or fields of cells (Figure 1D and 1E), implying there could be a ‘fourth’ pathway where the two proteins are co-expressed (n=10 out of 106 patients). Because these patient numbers come from tissue cores, obtained from the much a larger resected tumour mass, the cores cannot capture the true depth of heterogeneity of any one given cancer. Therefore, this figure is meant to be a working model to test the concept of mutually exclusive GPA33 and Claudin18.2 protein expression. Testing this would require IHC evaluation of the entire cancer tissue block, because it is possible that even if there are GPA33 and a Claudin18.2 mutually exclusive developmental paths, any one cancer could express both pathways in different regions of the tumour mass due to heterogeneity of cancer evolution. On the other hand, the lymph node metastatic sites arise from the primary tumour. Concordance between the primary and involved lymph node, with respect to GPA33 and a Claudin18.2 mutually exclusive expression (as in Supplementary Figure 2) would suggest that a primary tumour mass might not have heterogeneity between the two pathways.*

*SF2.*

*
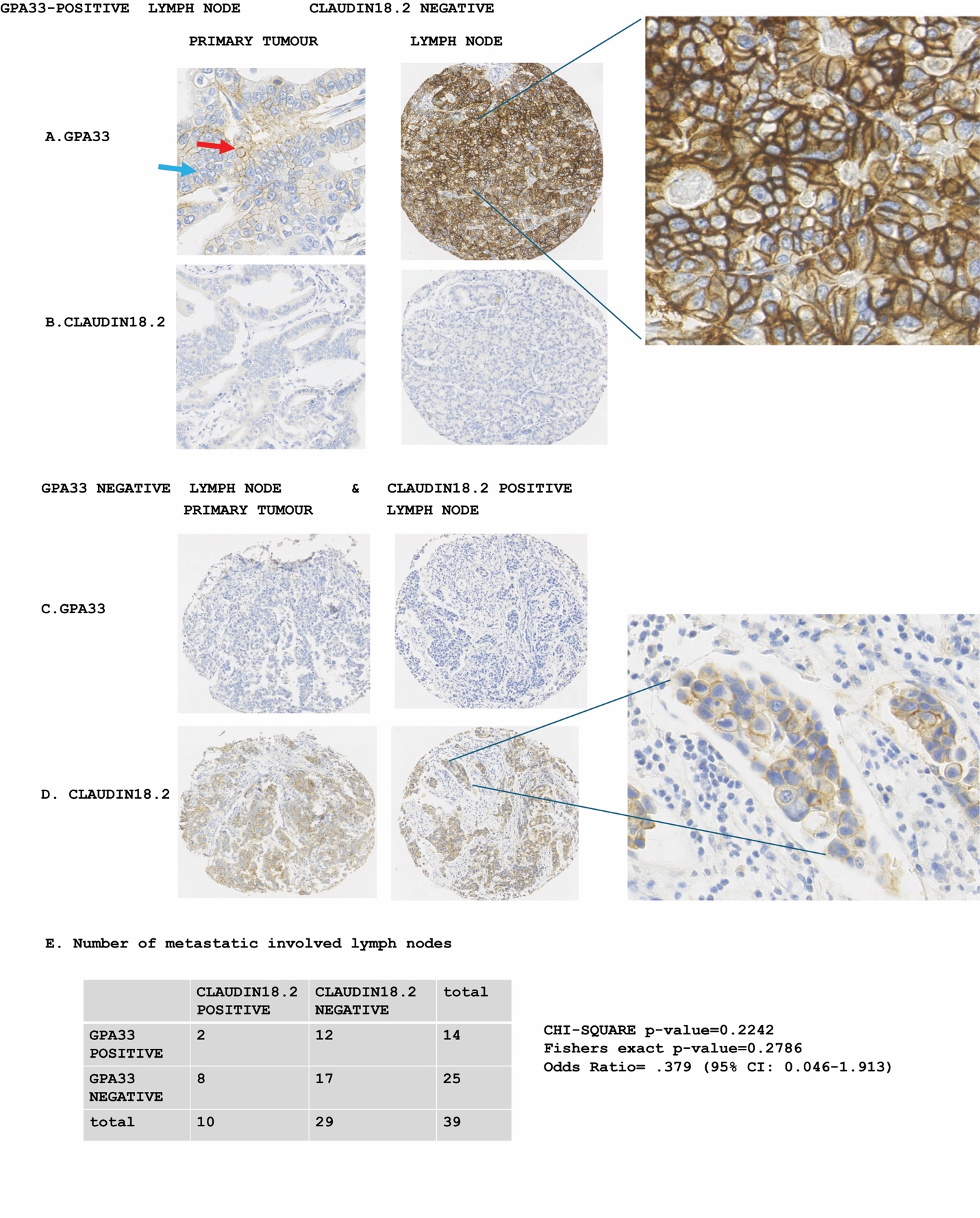
*

***SFigure 2. Representative expression of GPA33 and Claudin18.2 in lymph nodes****. A TMA containing adenocarcinoma, normal adjacent squamous oesophagus and gastric tissue, and cores from involved lymph nodes [20], was used to examine GPA33 and Claudin18.2 expression in involved lymph nodes to measure extent of metastatic dissemination. Serial slices of the TMA were taken to compare expression of GPA33 and Claudin18.2 in the same tissue region. A and B. Representative images with expression of GPA33 in primary tumour and matched involved lymph node, but no expression of Claudin18.2. The red and blue arrows in “A” highlight, within the same tumour field, membrane positive and membrane negative receptor expression, respectively. C and D. Representative image with expression of Claudin18.2 in primary tumour and matched involved lymph node, but no expression of GPA33. E. Quantitation of the extent of expression in GPA33 and Claudin18.2 in cores from n=39 different involved lymph nodes. The statistical data is as follows: Chi-square test (without correction): χ² = 1.477, df = 1, p = 0.2242; Fishers exact test:p = 0.2786; Odds Ratio = 0.379 (95% CI: 0.046 – 1.913). The conclusion is that is that the mutually exclusive expression of GPA33 and Claudin18.2 in lymph nodes is not statistically significant, despite the trend observed is similar to that in the primary cancers.*

*SF3*

*
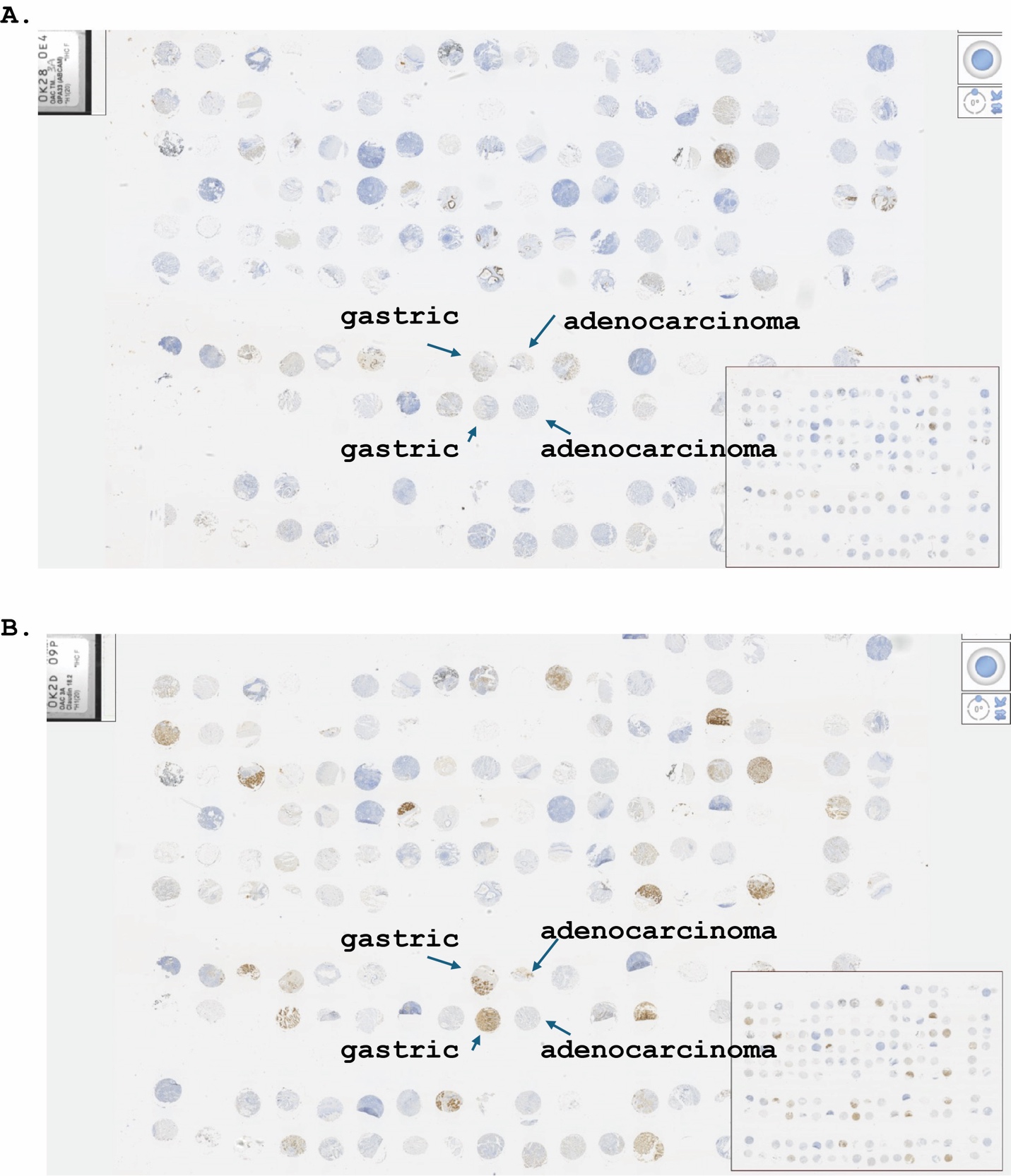
*

***SFigure 3. Representative macro view of immunohistochemistry in one TMA for (A). GPA33 and (B). Claudin18.2.*** *The TMA contains cores from adenocarcinoma, normal adjacent squamous oesophagus, involved and non-involved nodes, and normal adjacent gastric tissue [20]. Samples of gastric tissue and adenocarcinoma are highlighted. The stronger brown color in (B), which is the Claudin18.2 antibody IHC, highlights the high target expression in normal gastric tissue. Samples were stained as indicated in the materials and methods. The macroimages are shown along with the inset zoom view.*

*SF4.*

*
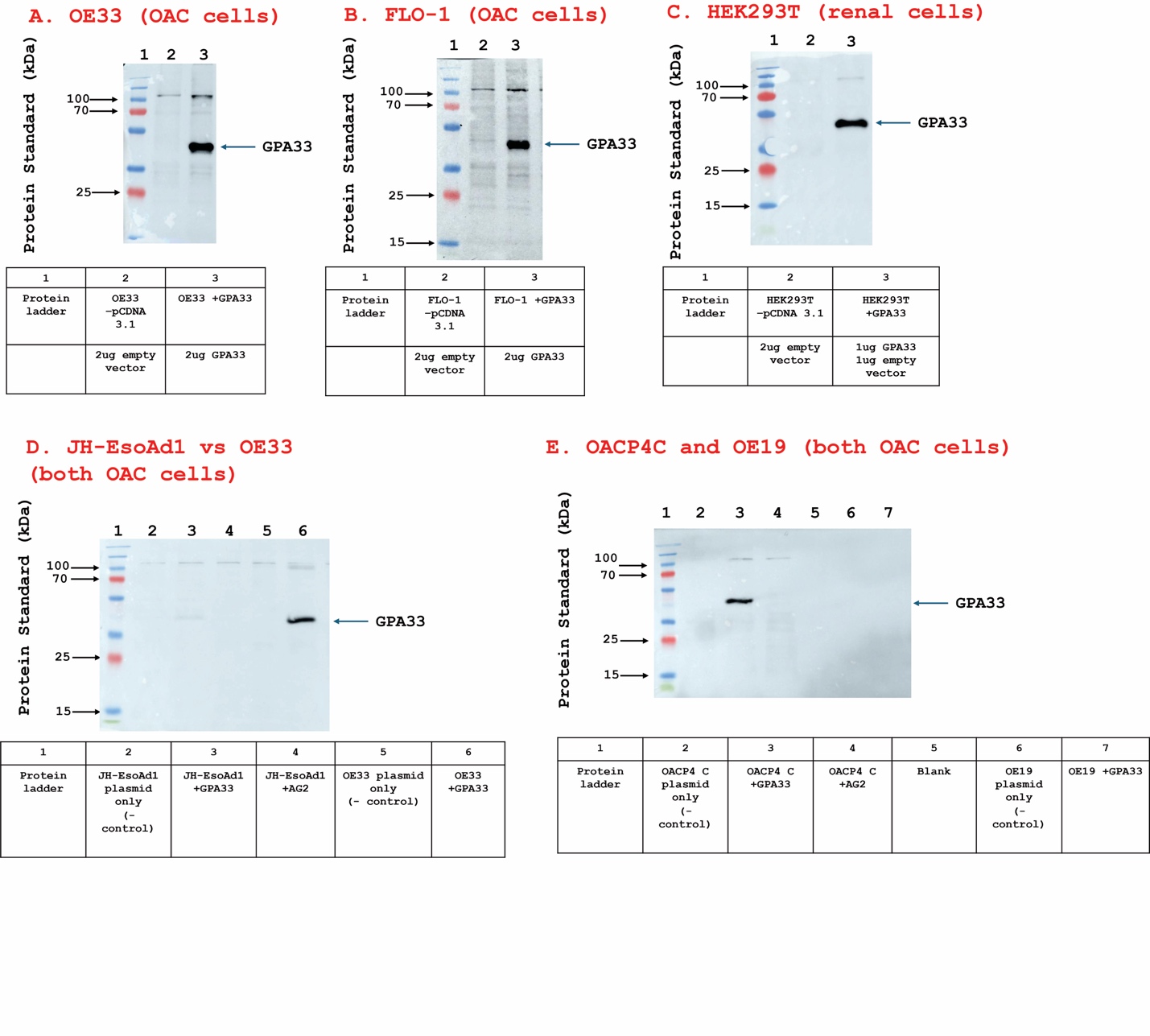
*

***SFigure 4. Examination of basal GPA33 protein in commonly used oesophageal cancer cell lines.*** *The indicated cell lines were transfected with vector plasmid only control or the GPA33 expression plasmid, as indicated. Some cell panels were also transfected with an AGR2 expression plasmid (as in D and E) which is not relevant for this study. The blots were probed using RSE-05 monoclonal antibody.*

*SF5.*

*
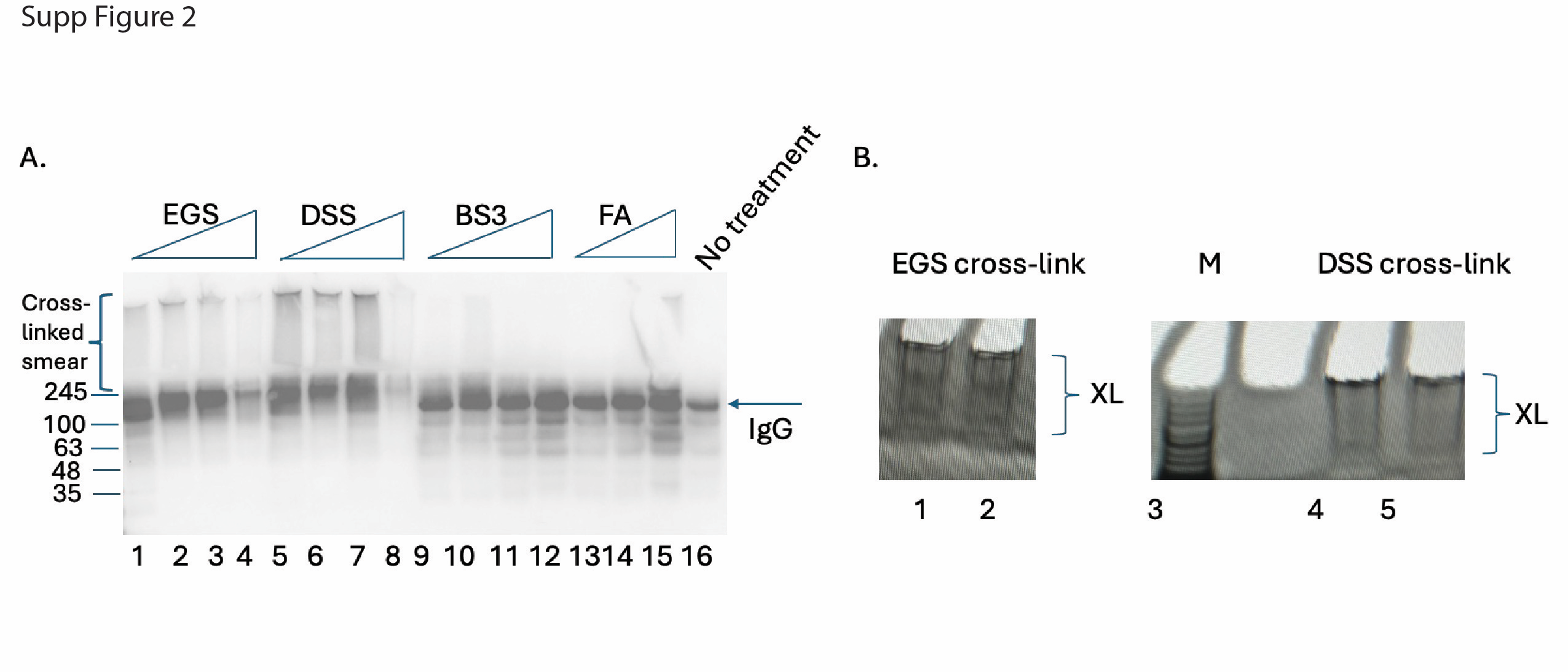
****SFigure 5. Mapping interfaces in the RSE-05 IgG:GPA33 protein complex using cross-linking mass spectrometry.*** *(A). Cross-linking tests. 100 ng of RSE-05 and 100 ng of his-tagged GPA33 were incubated in 20 ul of PBS. The indicated cross-linking agents were added) at concentrations of 15 mM, 3 mM, 0.6 mM and 0.15 mM) and incubated for 30 minutes. The reactions were quenched with LDS sample plus 0.2 M DTT, but the samples were not heated prior to denaturing gel electrophoresis. As a result, the IgG migrates at ~ 150 kDa mass. The gel was immunoblotted and probed with anti-mouse IgG to measure the extent of cross-linking as defined by reduced mobility and/or a smear (brackets) on the immunoblot. DSS gave rise to the most pronounced cross-linking and DSS was used to scale up the reaction for processing by cross-linking mass spectrometry (See Figure 8). Neither BS3 nor formaldehyde (FA) gave rise to notable cross-linking. (B) Scale up of the cross-linking reactions. RSE-05 (4.5 ug) and his-tagged GPA33 (6 ug) was prebound for 30 minutes in 40 ul of PBS and then 5 mM DSS or EGS was added for 30 minutes. The reactions were quenched with LDS sample plus 0.2 M DTT, but the samples were not heated prior to denaturing gel electrophoresis. Electrophoresis was carried out for 10 minutes followed by staining with Coomassie blue to identify the cross-linked smear, which was excised and processed for trypsinization as indicated in the Materials and Methods. Only the DSS cross-linked samples were processed for analysis (Figure 8).*

*SF6.*

*
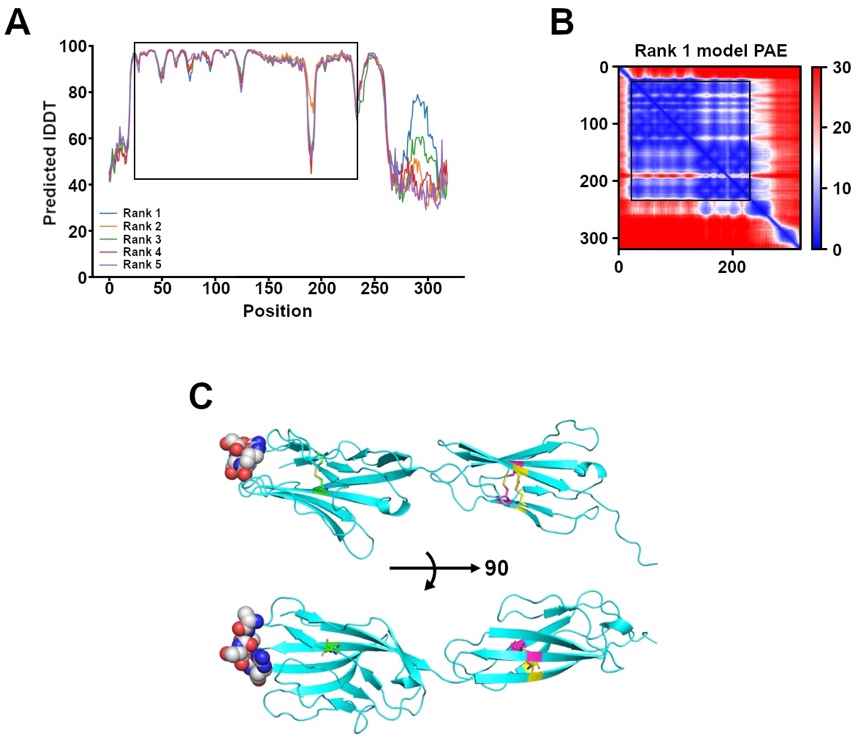
*

***SFigure 6. A structural model of GPA33 was constructed using ColabFold to facilitate the selection of complementary GPA33 two-site detection epitopes.*** *(A) Predicted lDDT for the top 5 models generated by ColabFold. The extracellular domain (residues 22-235) is indicated by the black box. B) Predicted Aligned Error for the rank 1 model. The extracellular domain is indicated within the black box. C) The model of GPA33 is shown with the disulphide bonds (yellow, green, magenta) and the STSSR motif (spheres). Two regions distal to the STSSR epitope of RSE-05 were selected for generation of protein-derived peptides for the second round of anti-GPA33 polyclonal antibody discovery (Figure 9).*

*SF7.*

*
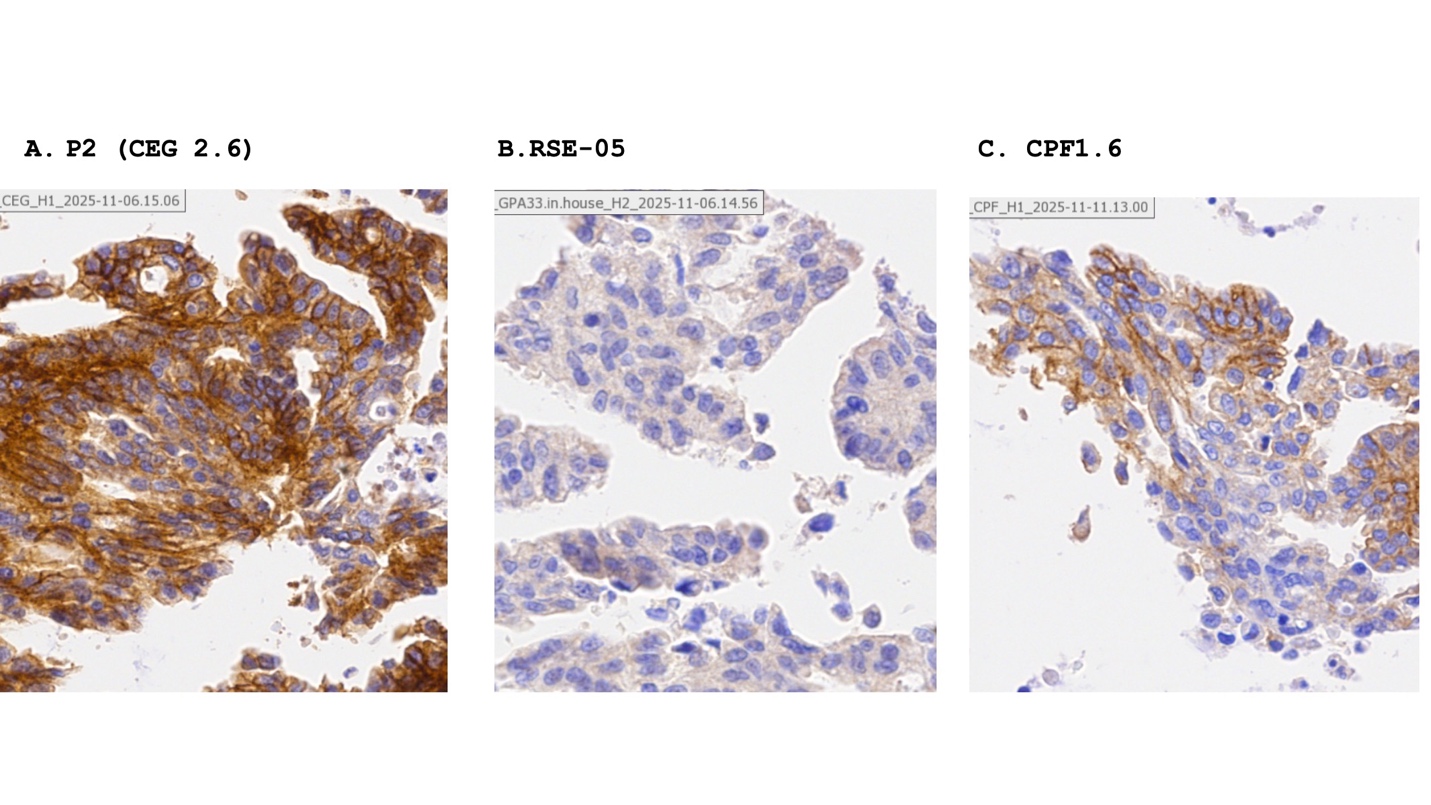
*

***SFigure 7. A TMA containing OAC was used to compare the ability of three antibodies to function in IHC vs ELISA (Figure 9).*** *The CEG2.6 and RSE-05 antibodies were used at 1:100 dilution, and the CPF1.6 antibody at 1:800 dilution, as indicated in the Materials and Methods and the images are representative. (A) P2 antibody (CEG2.6) which only binds well to DTT treated GPA33 (Figure 9C) binds to membranous material. (B). RSE-05 which does not bind to DTT treated GPA33 does not bind using IHC. (C). P1 antibody (CPF1.5) which binds well to either oxidized or DTT treated GPA33 (Figure 9C) binds to membranous material with regions of negative staining indicating heterogeneous pools of GPA33 exist in the same tissue, with respect to this epitope.*

*Supplementary Immunoblots*

*Original blots from Figure 3F including marker positions*

*
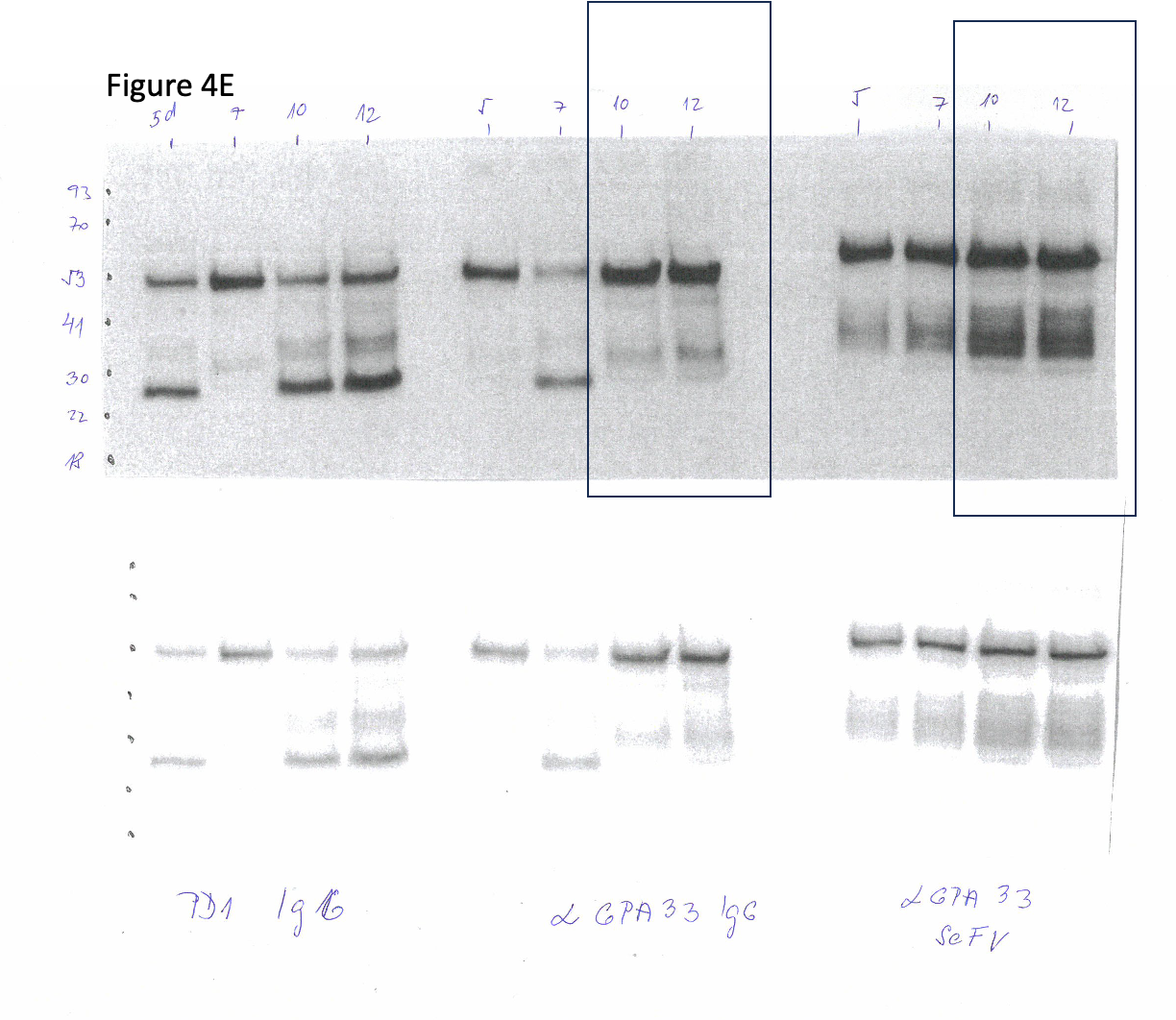
*

*Original blots from Figure 4A including marker positions.*

*
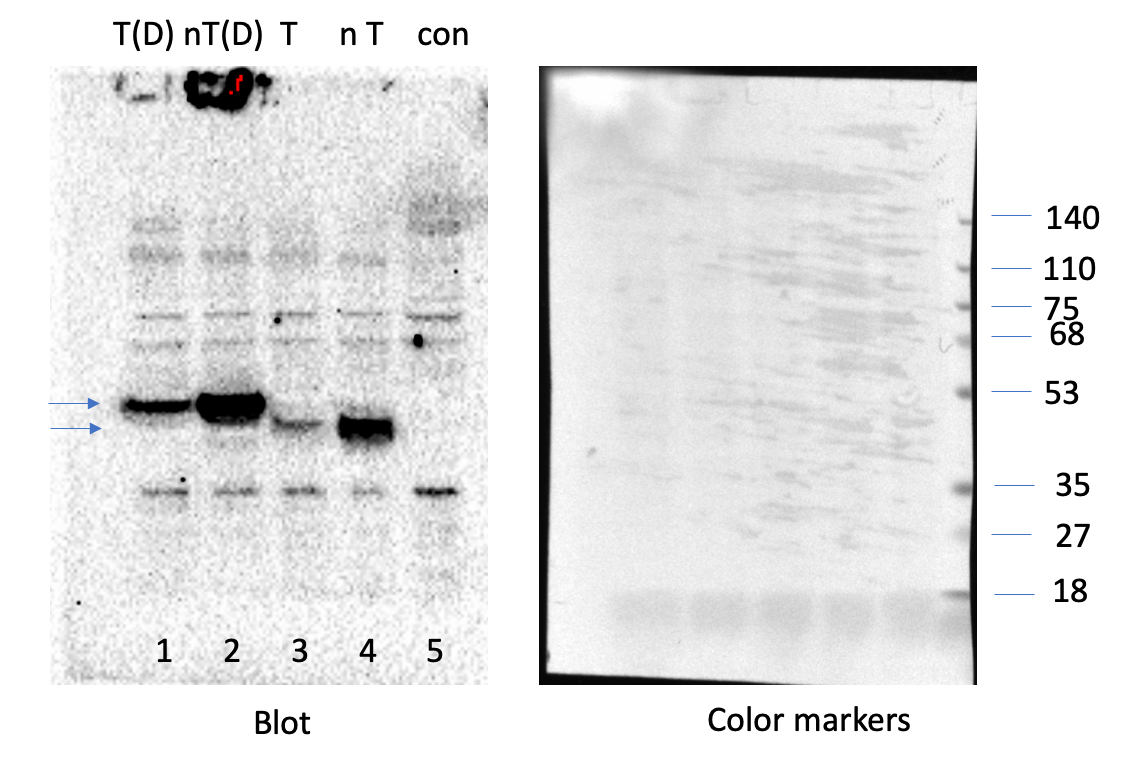
*
